# UGbS-Flex, a novel bioinformatics pipeline for imputation-free SNP discovery in polyploids without a reference genome: finger millet as a case study

**DOI:** 10.1186/s12870-018-1316-3

**Published:** 2018-06-15

**Authors:** Peng Qi, Davis Gimode, Dipnarayan Saha, Stephan Schröder, Debkanta Chakraborty, Xuewen Wang, Mathews M. Dida, Russell L. Malmberg, Katrien M. Devos

**Affiliations:** 1 Institute of Plant Breeding, Genetics and Genomics (Department of Crop and Soil Sciences), University of Georgia, Athens, GA 30602 USA; 2 Department of Plant Biology, University of Georgia, Athens, GA 30602 USA; 3 ICRISAT-Nairobi, P.O. Box 39063-00623, Nairobi, Kenya; 4 Institute of Bioinformatics, University of Georgia, Athens, GA 30602 USA; 5 Department of Genetics, University of Georgia, Athens, GA 30602 USA; 6 Department of Applied Plant Sciences, Maseno University, Maseno, Kenya; 7 Present address: Institute of Plant Breeding, Genetics and Genomics (Department of Horticulture), Tifton, GA 31793 USA; 8 Present address: ICAR-Central Research Institute for Jute and Allied Fibres, Kolkata, India; 9 Present address: Department of Plant Sciences, North Dakota State University, Fargo, ND 58108 USA

**Keywords:** Chromosomal rearrangements, *Eleusine coracana*, *E. indica*, Finger millet, Genetic mapping, Genotyping-by-sequencing (GBS), GBS-pipeline, Paired-end reads, Polyploid, SNP calling

## Abstract

**Background:**

Research on orphan crops is often hindered by a lack of genomic resources. With the advent of affordable sequencing technologies, genotyping an entire genome or, for large-genome species, a representative fraction of the genome has become feasible for any crop. Nevertheless, most genotyping-by-sequencing (GBS) methods are geared towards obtaining large numbers of markers at low sequence depth, which excludes their application in heterozygous individuals. Furthermore, bioinformatics pipelines often lack the flexibility to deal with paired-end reads or to be applied in polyploid species.

**Results:**

UGbS-Flex combines publicly available software with in-house python and perl scripts to efficiently call SNPs from genotyping-by-sequencing reads irrespective of the species’ ploidy level, breeding system and availability of a reference genome. Noteworthy features of the UGbS-Flex pipeline are an ability to use paired-end reads as input, an effective approach to cluster reads across samples with enhanced outputs, and maximization of SNP calling. We demonstrate use of the pipeline for the identification of several thousand high-confidence SNPs with high representation across samples in an F_3_-derived F_2_ population in the allotetraploid finger millet. Robust high-density genetic maps were constructed using the time-tested mapping program MAPMAKER which we upgraded to run efficiently and in a semi-automated manner in a Windows Command Prompt Environment. We exploited comparative GBS with one of the diploid ancestors of finger millet to assign linkage groups to subgenomes and demonstrate the presence of chromosomal rearrangements.

**Conclusions:**

The paper combines GBS protocol modifications, a novel flexible GBS analysis pipeline, UGbS-Flex, recommendations to maximize SNP identification, updated genetic mapping software, and the first high-density maps of finger millet. The modules used in the UGbS-Flex pipeline and for genetic mapping were applied to finger millet, an allotetraploid selfing species without a reference genome, as a case study. The UGbS-Flex modules, which can be run independently, are easily transferable to species with other breeding systems or ploidy levels.

**Electronic supplementary material:**

The online version of this article (10.1186/s12870-018-1316-3) contains supplementary material, which is available to authorized users.

## Background

Efficient genotyping methods, whether used for mapping or population genetic studies, must be simple and reliable, and provide the allele composition at thousands or more of polymorphic loci that cover the entire genome [1]. Despite the recent advances in sequencing technologies, whole genome sequencing is still not cost effective for large-genome species, especially when multiple-fold coverage needs to be achieved of several hundred individuals. Several reduced representation methods based on selective sequencing of restriction fragments have been developed to simultaneously conduct high-throughput marker discovery and genotyping [2–4]. We collectively refer to these methods as ‘genotyping-by-sequencing’ (GBS). To keep sequencing costs low, there is typically a trade-off between marker number and sequencing depth. As a result, genotyping-by-sequencing data sets often have large amounts of missing data and low sequence coverage at each locus [2, 5, 6]. Imputations can be used to infer missing genotypes [7]. Low sequence depth, however, is highly problematic when analyzing diversity panels in outcrossing species, and biparental backcross, F_1_ (in outcrossing species) and F_2_ populations where sufficient sequence depth at each locus is a prerequisite to unambiguously differentiate homozygous and heterozygous alleles. We therefore implemented several modifications to the experimental GBS protocol developed by Elshire et al. [2] and Poland et al. [8], and tested their effect on reducing the GBS fragment pool and providing more even read coverage across pooled samples for high-confidence imputation free single nucleotide polymorphism (SNP) identification.

Analysis of GBS reads is fairly straightforward if whole genome sequence data is available to which the GBS reads can be aligned. Pipelines for reference-based GBS analysis include TASSEL-GBS [9, 10], Fast-GBS [11] and Stacks [12]. Pipelines such as TASSEL-UNEAK [5], Stacks [12] and GBS-SNP-CROP [13] can generate a *de novo* GBS reference from the experimental data and are aimed at the analysis of GBS data from species without reference genomes. Most pipelines are geared towards single-end sequencing data from diploid organisms.

Finger millet, *Eleusine coracana* (L.) Gaertn. subsp. *coracana*, is an inbreeding allotetraploid (AABB) cereal belonging to the subfamily Chloridoideae with a haploid genome size of 1.7 Gb [14]. Despite being an important food security crop for Eastern Africa and parts of southern India, it has persistently been neglected by the international research community. The wild progenitor of finger millet, *E. coracana* subsp. *africana*, originated by hybridization between *E. indica* (AA genome) and an unknown B-genome species. The timing of the allopolyploidization event is not known. To date, only a single linkage map has been generated of finger millet consisting of 332 loci, mostly detected by restriction fragment length polymorphism (RFLP) markers and single strand conformation polymorphic expressed sequenced tags [15, 16]. The map was generated in an F_2_ population derived from a cross between a wild accession, MD-20, and a cultivated accession, Okhale-1. Based on similarity between RFLP fragment sizes in the A-genomes of *E. indica* and *E. coracana*, linkage groups were allocated to subgenomes [15]. The linkage map was also used to establish gross comparative relationships between the finger millet and rice genomes [16]. There is a need for high-density SNP maps of finger millet to assist with trait analyses and planned genome sequencing efforts.

Even with less than 10,000 markers, construction of an accurate linkage map is extremely challenging, in particular when dealing with less than perfect data sets such as, for example, obtained in F_3_-reconstituted F_2_ populations. Traditional software like MAPMAKER [17, 18] and JoinMap [19, 20] use maximum likelihood based three-point and multi-point analyses which provide highly accurate marker ordering but are highly memory- and time-intensive for large data sets. To deal with large marker numbers, software packages such as MSTmap [21] and Lep-Map [22] based on the traveling salesman principle have been developed. Map generation is fast, but marker ordering is more sensitive to genotyping errors. To be able to take advantage of MAPMAKER’s high accuracy for ordering large marker sets, we modified the original MAPMAKER package to run efficiently in a Windows Command Prompt Environment and developed in-house python scripts to automate several steps of the MAPMAKER mapping process. The mapping pipeline was applied to generate a new high-density genetic map of finger millet comprised of several thousand high-quality SNP markers.

Our paper thus provides a modified GBS protocol, a new pipeline (UGbS-Flex) for analysis of paired-end GBS data suitable for application in species with different ploidy levels, breeding systems and polymorphism levels, irrespective of the availability of a reference genome sequence. We also provide a comprehensive solution for post-GBS data analysis, and a high-density genetic map of finger millet with new information on the organization of the allotetraploid finger millet genome.

## Methods

### Plant materials and DNA extraction

The F_2_ mapping population was generated from a cross between *E. coracana* subsp. *africana* accession MD-20 and *E. coracana* subsp. *coracana* accession Okhale-1 [15]. One hundred and thirty-four F_2:3_ families with a minimum of 15 plants per F_3_ family plus the parents were grown in the greenhouse at UGA under day/night temperatures in the range 26–30 °C. KNE 796, an accession for which whole genome sequencing is ongoing, and *E. indica* accessions Ei-0, Ei-2 and Ei-5, collected in the wild in Kenya, were grown under the same conditions. All germplasm was obtained in compliance with national and international accords on export/import of seeds for research purposes. DNA was extracted using a modified CTAB method adapted from Doyle and Doyle [23] from leaves bulk-harvested for each F_3_ family from eight weeks old seedlings. DNA concentrations were measured on a Nanodrop (Thermo Scientific) and samples were diluted to 50 ng/μl. The quality of the DNA was assessed on a 0.8% agarose gel.

### GBS sample preparation

Two-hundred nanogram of high-molecular-weight DNA was digested with a cocktail of either *Pst*I/*Msp*I, *Pst*I/*Nde*I or *Pst*I/*Msp*I + *Ape*KI. Digestions were done in volumes of 30 μl with 4 U *Pst*I-HF and 8 U *Msp*I in NEB CutSmart buffer for *Pst*I/*Msp*I digestions, 4 U *Pst*I, 8 U *Msp*I and 4 U *Ape*KI in NEB-buffer 3.1 for *Pst*I/*Msp*I plus *Ape*KI digestions, and 4 U *Pst*I-HF and 4 U *Nde*I in NEB CutSmart buffer for *Pst*I/*Nde*I digestions. Samples were incubated for 2 h at 37 °C. This was followed by an additional 2-h incubation at 75 °C for reaction mixtures comprising the enzyme *Ape*KI. Samples without *Ape*KI were incubated at 75 °C for 20 min to inactivate the restriction enzymes.

Twenty microliter restriction digest was mixed with 1 μl barcoded *Pst*I adapter (stock: 0.1 μM), 1.5 μl common *Msp*I or *Nde*I Y-adapter (stock: 10 μM), 4 μl 10X T4-DNA ligase buffer and 200 U T4 DNA ligase (NEB) in a total volume of 40 μl. The common Y-adaptor and barcoded adapters were as described by Poland et al. [8]. Ligation was conducted at 22 °C for 2 h. Following ligation, fragments smaller than 300 bp were removed by incubating the samples with 0.7 volumes of Sera-Mag SpeedBeads (GE Healthcare Life Sciences) prepared according to Rohland and Reich [24] at room temperature for 5 min. The beads were separated from the supernatant using a magnetic stand and washed three times with 200 μl freshly prepared 70% ethanol. DNA was eluted from the air-dried beads with 40 μl 10 mM Tris.HCl (pH 8.0).

Three microliter of the resulting eluate was added to a cocktail of 16 μl H_2_O, 5 μl 5X Taq master mix (NEB), 0.5 μl of a forward primer specific to the barcoded adaptor (stock: 10 μM) and 0.5 μl of a reverse primer with homology to the common adaptor (stock: 10 μM). PCR amplification was done for each sample separately using an initial denaturation at 95 °C for 30 s, 16 cycles of denaturation at 95 °C for 30 s, primer annealing at 62 °C for 20 s and fragment elongation at 68 °C for 15 s, followed by a final fragment elongation step at 68 °C for 5 min. Eight microliters of PCR product were checked on a 1.5% agarose gel. The DNA concentration of each GBS library was measured on a Qubit 2.0 using a Qubit™ dsDNA HS assay kit. Only GBS libraries with concentrations > 5.0 ng/μl were sequenced. Thirty nanograms of each GBS library were pooled. The number of samples pooled depended on the sequencing platform used; we aimed to obtain two million reads per sample. Primers, dNTP and small DNA fragments were removed from the pooled DNA with 0.7 volumes of AMPure Beads or Sera-Mag SpeedBeads. Pooled GBS libraries (100 ng) were sequenced on an Illumina NextSeq platform with paired-end 150 bp reads. The parents and 115 F_2:3_ samples were sequenced as part of the same sequencing run. An additional three and 26 F_2:3_ samples were sequenced as part of two separate NextSeq runs. Ten samples were sequenced in duplicate from independently generated libraries to ensure consistency across libraries and runs.

### *GBS analysis pipeline with optional* de novo *generation of a reference*

The full UGbS-Flex pipeline is described below. All in-house perl and python scripts used in the UGbS-Flex pipeline with information on their use are provided under ‘Programs and Scripts’ on http://research.franklin.uga.edu/devoslab/. Detailed information on how to apply the UGbS-Flex pipeline is given in Additional file 1: Data S1 and Additional file 2: Figure S1.

#### Preprocessing of the reads

The read quality was checked with ‘FastQC’ v. 0.11.4 [25]. Reads were split by barcode using the module ‘Process_Radtags’ within the ‘Stacks’ program [12] with option –r (rescue barcodes and RAD-tags). Forward reads passed the filter if they carried both the barcode and the *Pst*I restriction site. Because the first few bases of the reverse reads were low quality in some Illumina NextSeq sequencing runs, no selection was carried out for the restriction site of the second enzyme (*Msp*I or *Nde*I) in the reverse reads. ‘FASTX_trimmer’ from the ‘FASTX Toolkit’ package (http://hannonlab.cshl.edu/fastx_toolkit/) was used to remove (1) the restriction sites, (2) the last 5 bp (typically) of each read that were more likely to contain errors (or, for lower quality runs, all bases at the 3′ end of a read with FastQC quality scores lower than 20) and (3) an additional 0 (for a 10 bp barcode) to 5 bases (for a 5 bp barcode) at the 3′ end of the forward read to make all reads the same length. Identical read length is a prerequisite of the ‘Stacks’ program [12] used in the generation of a *de novo* reference from the GBS reads.

#### *De novo* generation of a GBS reference

**Fig. 1:**
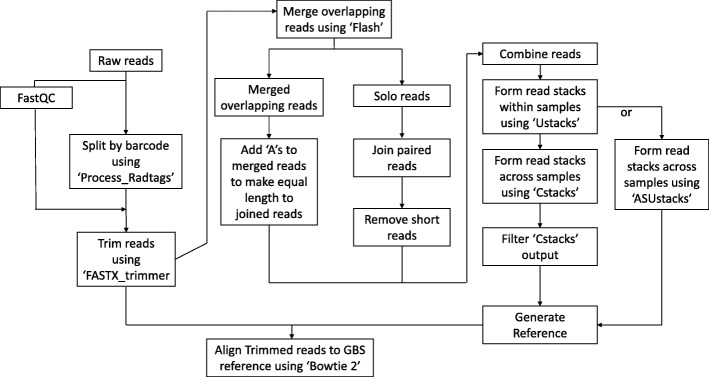
Schematic overview showing use of the UGbS-Flex pipeline to generate a GBS reference

To facilitate handling of paired-end reads during the generation of a GBS reference, overlapping forward and reverse reads were merged using ‘Flash’ [26]. From the non-overlapping read files, we removed any reads that were shorter than the expected (trimmed) size by running python script ‘EL.1.2.py’. If a reverse read was removed, the corresponding forward read was also removed, and *vice versa*. The ‘EL.1.2.py’ script then reverse complemented the non-overlapping reverse reads within the remaining read pairs and artificially joined them to the 3′ end of the corresponding forwards reads. No Ns were added at the junction of the forward read and the reverse complemented reverse read. Because read clustering by ‘ustacks’ [12] requires reads of equal length, ‘EL.1.2.py’ also extended merged overlapping reads at the 3′ end with ‘As’ to make them the same length as the joined non-overlapping reads. The A-extended overlapping fragments are typically common to all samples, and hence the polyA tracts do not generate polymorphisms. Reads within each sample were clustered using the ‘ustacks’ module (options: -m 2 -M 1 -N 1) within the ‘Stacks’ program [12]. The ‘cstacks’ module within Stacks (options: -b 1 -n 1) was used to generate a set of representative tags by clustering the read stacks obtained from ‘ustacks’ across the two parents and 117 F_2_ progeny. Only a subset of the F_2_ samples was included in the ‘cstacks’ analysis due to the high memory requirements for running ‘cstacks’ with large numbers of samples. We also tested and validated an alternative approach, referred to as ‘across-sample ustacks’ (‘ASustacks’) to replace ‘cstacks’. Using in-house python scripts, consensus sequences generated in each sample by ‘ustacks’ were extracted, given a unique name including a sample identifier, and placed in an artificial fastq file by adding a sequence quality line consisting of Es to each consensus sequence. The ‘ustacks’ module was then applied to this file using parameters comparable to those applied in ‘cstacks’. The minimum number of reads required to form a stack (−m) was set at 1. An overview of the steps involved in the generation of a GBS reference using the UGbS-Flex pipeline is shown in Fig. 1.

#### Filtering of the GBS representative tags to generate a reference

Two filtering steps were conducted on the representative tags identified across samples by either ‘cstacks’ or ‘ASustacks’ (Fig. 1). Firstly, representative tags that were present in less than a user-defined percentage of samples (50 and 70% in this study) in the ‘cstacks’ output were removed using the in-house perl script ‘FCT.pl’. This filtering step has been integrated into the ‘ASustacks’ module. Secondly, we removed representative tags that had similarity levels to another representative tag equal to or higher than a user-defined percentage (98% in this study). To achieve this, a blastn all-against-all analysis of the consensus tags was conducted (e-value threshold: 10e^− 5^). For tags with ≥98% homology, only a single tag was retained using python script ‘Ref_98.py’. The representative tags remaining were used as the GBS reference.

#### SNP/indel calling and filtering

Preprocessed reads were aligned against the GBS reference using Bowtie 2 v. 2.2.0 with default parameters [27]. If a whole genome reference sequence is available, *de novo* generation of a GBS reference can be omitted and trimmed reads are aligned against the reference genome. For SNP/indel calling, we tested both the ‘Unified Genotyper (GATK v. 3.4.0)’ (parameters –dcov 1000, −glm BOTH) and ‘Haplotype Caller (GATK v. 3.4.0)’ (parameters --genotyping_mode DISCOVERY –stand_emit_conf 10 –stand_call_conf 30 –minPruning 1 –emitRefConfidence GVCF) modules within the GATK suite [28]. Indels were treated the same as SNPs and, for simplicity, we use the term ‘SNPs’ to cover both SNPs and indels. Raw SNPs were filtered within GATK to only retain biallelic SNPs with an allele frequency between 10 and 90%. We also removed adjacent SNPs (scripts ‘SNPs_ISL.pl’ and ‘Rm_adj_SNPs.pl’) because some of these were artefacts caused by misalignments at the junction of the forward and reverse reads. The allelic depth (AD) information provided in the GATK .vcf file was then used to score the allelic status of the SNPs at each locus (script ‘SNP_genotyper.py’). Loci with a total AD < 8 were scored as missing data points (−). Loci with an AD_ref(erence allele)_/AD_alt(ernate allele)_ ratio ≥ 10 were scored as A (homozygous for the Parent 1 allele), AD_ref_/AD_alt_ ≤ 0.10 as B, 10 > AD_ref_/AD_alt_ > 4 as D (ambiguous A or heterozygous (H)) and 0.25 > AD_ref_/AD_alt_ > 0.1 as C (ambiguous B or H). Loci with other ratios were scored as H. For F_2_ populations generated from two inbred parents, as was the case for finger millet, all SNPs that were not homozygous for different alleles in the parents were removed (script ‘SNP_selectByParent.py’). SNPs that were missing in more than 30% of the samples or had an A/B ratio < 10% or > 90% were also removed. This was done manually in Excel. Similarly, samples with more than 30% of missing data were removed.

#### Comparison of the UGbS-flex and GBS-SNP-CROP pipelines

To compare the performance of the UGbS-Flex and GBS-SNP-CROP [13] pipelines, the raw sequence data from 48 tetraploid kiwiberry genotypes used by Melo et al. [13] were downloaded from NCBI (SRR2296676). Guided by the fastQC report, we trimmed the forward and reverse reads to 121 bp. The parameters used for ‘ustacks’ were –m 2 –M 2 –N 4, and for ‘ASustacks’–m 1 –M 2 –N 4. We selected the same missing data threshold (25%) and sequencing depth for SNP scoring as Melo and colleagues [13]. The H-threshold was set at 4.

### Genetic mapping

#### Reducing the dataset by consolidating SNPs located in the same GBS tag

Information at SNP loci that were located within the same GBS reference tag and were in linkage disequilibrium was consolidated to further improve the robustness of the mapping scores (function included in ‘SNP_genotyper.py’). If loci within a representative tag were scored as a combination of ‘A’, ‘D’ and ‘-’, the consolidated score was ‘A’ (Table 1). Similarly, a combination of ‘B’, ‘C’ and ‘-’ was consolidated as ‘B’, and a combination of ‘H’,‘C’, ‘D’ and ‘-‘ as H. If all loci in a GBS representative tag were scored as ‘-’, ‘C’ or ‘D’, those scores were retained. Conflicting scores (A and B, A and C, A and H, B and H, and B and D) were flagged (‘F’) and treated as missing data for map generation.

**Table 1:** Schematic representation of the approach used to consolidate SNPs within the same GBS reference tag

	S1^b^	S2	S3	S4	S5	S6	S7	S8	S9	S10	S11	S12
SNP1^a^	–	B^c^	–	C	D	A	B	H	A	B	H	A
SNP2^a^	A	–	–	–	–	D	C	C	A	D	A	A
SNP3^a^	–	–	H	–	–	A	C	D	C	B	A	B
After consolidation	**A**	**B**	**H**	**C**	**D**	**A**	**B**	**H**	**–**	**–**	**–**	**–**

^a^SNP1, SNP2 and SNP3 are SNPs located on the same GBS tag

^b^S1 to S12 represent different situations

^c^A, B, H, C, D and – are genotypic scores

#### Reducing the dataset by removing cosegregating markers

To generate a set of high-quality non-redundant markers for genetic map construction, each SNP marker was given a penalty score for the presence of a ‘C’ or ‘D’ (penalty = 1) or a missing data point (penalty = 2). Using the in-house python script ‘SNP_cosegregation.py’, the mapping scores of all SNP markers were compared in an all-against-all analysis with a greedy algorithm. SNP markers with the same multilocus genotypes were identified, and the marker with the smallest penalty score in each set was selected for mapping.

#### Construction of genetic maps

To construct the genetic maps, we removed all SNPs that had missing data in more than 20% of the progeny. The set of SNP markers was split into linkage groups using MSTmap [21]. Initial map orders were established with MST map and checked for double recombination events using MAPMAKER (adapted from [18]; adapted version available from http://research.franklin.uga.edu/devoslab/)*.* Markers with more than a defined number (four in this study) of double recombination events were removed. The process of MSTmap mapping and checking of double recombination events was repeated until the number of double recombination events between each marker and its flanking markers was ≤4. The corresponding MSTmap maps were used as starting points for map generation using MAPMAKER (adapted from [18]; adapted version available from http://research.franklin.uga.edu/devoslab/).

Because the MAPMAKER version used was limited to ordering ~ 100 markers due to inherent program limitations, MSTmap maps with more than 100 markers were split into smaller subgroups of 60 to 100 markers. Subgroups overlapped by 40 markers. Genetic maps were constructed for each of the subgroups using the ‘order’ and ‘try’ commands. Subgroup maps were merged based on common marker orders in the overlapping segments. Each linkage group was split again into subgroups of < 100 markers, and marker orders were further adjusted using the ‘ripple’ command. ‘Try’ and ‘ripple’ were done in a semi-automated manner using the scripts ‘try.py’ and ‘ripple.py’, respectively. Final marker orders were merged across subgroups. Genetic map distances (in Kosambi) were obtained using the ‘map’ command with ‘error detection on’ in MAPMAKER. Map orders were scrutinized manually and, if necessary, further adjusted. Markers with the same multi-locus genotype were added to the map as cosegregating with their representative marker. Finally, we placed markers with ambiguous orders (not separated by clear recombination events) in bins.

### Identifying a and B genome linkage groups

Reads from three *E. indica* accessions (AA genome) were aligned against the GBS reference tags using Bowtie 2 v. 2.2.9 with default parameters [27]. Presence (present in at least two of the three *E. indica* accessions analyzed) and absence (absent from all three *E. indica* accessions analyzed) of mapped GBS tags in the *E. indica* genome were charted along the length of the genetic map using Excel scatterplots. Linkage groups with *E. indica* tags along their entire length were allocated to the A genome and those that were largely devoid of *E. indica* tags were allocated to the B-genome.

## Results

### Efficiency of different enzyme combinations in generating polymorphic markers

We tested two two-enzyme combinations (*Pst*I/*Msp*I and *Pst*I/*Nde*I) and one three-enzyme combination (*Pst*I/*Msp*I + *Ape*KI) on three finger millet accessions for their efficiency in generating largely overlapping fragment pools that, when sequenced, yielded SNPs that were present in all three accessions at a depth of at least 8×. All samples were sequenced (paired-end 150 bp) on an Illumina NextSeq platform. The number of reads obtained for each of the nine sample/enzyme combinations (three accessions, three enzyme combinations) is given in Additional file 3: Table S1. To estimate the effect of read depth on the *de novo* generation of a GBS reference, we analyzed subsets of 0.2 million (M), 0.5 M, 1 M, 2 M and 3 M paired-end reads for each accession/enzyme combination with our newly developed UGbS-Flex pipeline (Fig. 1). The smaller read numbers were subsets of the larger read sets.

For the enzyme combinations *Pst*I/*Msp*I and *Pst*I/*Msp*I plus *Ape*KI, as expected, the number of GBS tags that were common to all three accessions tested increased with increasing total read numbers, reaching a plateau around 2 M reads (Additional file 3: Table S1 ‘By Enzyme Combination’). For the enzyme combination *Pst*I/*Nde*I, however, the number of common GBS tags increased from 0.2 M to 1 M total reads, but then decreased when total read numbers were increased from 1 M to 3 M. To test whether this was an artefact generated by the ‘cstacks’ [12] module, which clusters reads across samples, we developed the script ‘across-sample ustacks’ (‘ASustacks’). ‘ASustacks’ generated an artificial fastq file from each sample’s ‘ustacks’ output and these files were used as input for ‘ustacks’. The ‘ASustacks’ approach yielded similar numbers of reference tags as ‘cstacks’ except for read numbers ≥1 M in enzyme combination *Pst*I/*Nde*I. We now saw the expected increase in common GBS tags with increasing read numbers across all enzyme combinations. More than 97% of GBS reference tags that were identified with ‘cstacks’ were also found in the reference generated using ‘ASustacks’. Interestingly, the read depth of the GBS reference tags that were identified by both ‘cstacks’ and ‘ASustacks’ was significantly lower than the read depth of the GBS reference tags that were uniquely identified by ‘ASustacks’ (Additional file 4: Table S2). This suggests that high read depth hampered the performance of ‘cstacks’, possibly because a higher read depth led to a higher absolute presence of SNPs caused by PCR or sequencing errors in allelic reads. The ‘cstacks’ module may have eliminated these clusters as likely consisting of repetitive DNA. We conducted a blastn analysis of all finger millet reference tags that were identified by ‘ASustacks’ in the subset of 3 M reads in the *Pst*I/*Nde*I digested samples against the repeat-masked rice (*Oryza sativa*) genome. Blastn hits were identified (e-value threshold of e-5) for 30% of the tags that were common to ‘cstacks’ and ‘ASustacks’, and for 37% of the tags that were uniquely identified by ‘ASustacks’. This shows that the tags eliminated by ‘cstacks’ were not enriched for repeats. To further validate the ‘ASustacks’ approach, we compared read clusters generated by ‘cstacks’ and ‘ASustacks’ in GBS data from DNA of a set of 96 diverse foxtail lily lines belonging to different *Eremurus* species digested with *Pst*I/*Msp*I. Foxtail lily has an ~ 7.9 Gb (1C) genome (I. Leitch, pers. comm.). The number of tags that were common to at least 50% of the lines based on ‘cstacks’ and ‘ASustacks’ was 376 and 3552, respectively, with 98% of ‘cstacks’ clusters being present in the ‘ASustacks’ output. In addition to yielding higher numbers of reference tags, the ‘ASustacks’ approach was computationally much less intensive than ‘cstacks’.

To estimate the effect of the different enzymes on reducing the fragment pool for sequencing, we compared the number of polymorphic GBS tags that were present in all three accessions tested and the number of SNPs identified across the three enzyme combinations (Table 2; Additional file 3: Table S1 ‘By Read Number’). Minimum read depth for SNP scoring was 8×. The use of a third enzyme greatly decreased the number of GBS reference tags and hence the number of SNPs identified but, contrary to our expectations, only marginally increased SNP read depth (Table 2). The percentage of *Ape*KI sites in the *Pst*I/*Msp*I read set was some two-fold higher than in the *Pst*I/*Msp*I + *Ape*KI read set, and comparable to that found in the GBS reference tags (Additional file 5: Table S3). In the triple digest, however, *Ape*KI-containing reads were highly underrepresented in the GBS reference compared to the reads (Additional file 5: Table S3). This suggests that *Ape*KI-containing reads in the triple digests could not be clustered. They likely originated during the adapter-ligation step through random ligation of *Ape*KI fragments from different genomic regions. The highest number of polymorphic SNPs present at a minimum read depth of 8× in each of the three accessions tested was obtained with enzyme combination *Pst*I/*Msp*I for read numbers ≥1 M and with *Pst*I/*Nde*I when the number of fragments sequenced per sample was ≤500,000 (Additional file 3: Table S1 ‘By Read Number’).

**Table 2:** Summary statistics obtained for each of the three enzyme combinations for a subset of 1 M reads

Enzyme combination	Accession	Total read number	Number of stacks (tags) within samples^a^	Number of stacks (tags) common to all samples^b^	Number of polymorphic tags^c^	Number of polymorphic SNPs^c^	Average depth of scored SNPs
*Pst*I/*Msp*I	KNE 796	5,720,433	83,082	22,191			15.20
	MD-20	5,418,738	64,382		4440	4515	15.64
	Okhale-1	7,738,023	73,903				17.21
*Pst*I/*Msp*I + *Ape*KI	KNE 796	3,466,582	50,189	12,249			16.57
	MD-20	4,474,070	37,619		2475	2509	20.90
	Okhale-1	5,586,989	45,829				19.65
*Pst*I/*Nde*I	KNE 796	6,647,185	39,406	12,392			32.28
	MD-20	4,269,463	35,218		3232	3305	28.66
	Okhale-1	5,851,494	37,551				34.76

^a^Determined using ‘ustacks’

^b^GBS tags that are common to all three accessions were selected from the ‘ASustacks’ output; if two or more tags had a level of homology ≥98%, only a single tag was retained

^c^Only SNPs at a read depth ≥ 8× were scored

### Generation of a GBS reference in the MD-20 x Okhale-1 mapping population

A total of 278,880,767 paired-end reads were obtained across 146 samples (SRA Study **SRP136342**). Genotypic scores of duplicated samples were merged using the rules applied to SNP consolidation. Approximately 2% of SNPs disagreed between duplicated samples and were entered as missing data. The average and median read number per sample was 1,910,142 and 1,317,595, respectively. One sample with less than 600,000 reads was removed from the analysis. The number of representative GBS tags that were present in at least 50% of the samples (hereafter referred to as Ref50) following ‘cstacks’ analysis was 34,960 (Table 3). This number decreased to 16,725 for tags present in at least 70% of the samples (Ref70). Following removal of representative tags with ≥98% homology, 28,579 tags remained in the Ref50 reference (Ref50_98) and 15,397 in the Ref70 reference (Ref70_98).

**Table 3:** Number of SNPs identified with different combinations of SNP callers and GBS references

	Unified Genotyper (GATK)				Haplotype Caller (GATK)			
	Ref50	Ref50_98	Ref70	Ref70_98	Ref50	Ref50_98	Ref70	Ref70_98
No. of GBS tags	34,960	28,579	16,725	15,397	34,960	28,579	16,725	15,397
No. of SNPs	2358	5534	3939	4477	1766	4593	3413	3934

### SNP calling

**Fig. 2:**
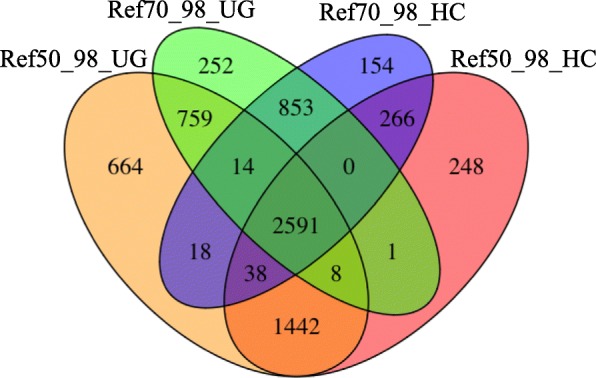
Venn-diagram showing the number of unique and common SNPs identified using GATK’s Unified Genotyper and Haplotype Caller in combination with GBS references Ref50_98 and Ref70_98

Trimmed reads were aligned against the generated Ref50, Ref50_98, Ref70 and Ref70_98 GBS references and the alignments were used for SNP calling. Both GATK’s Unified Genotyper and Haplotype Caller were employed and their outputs compared. The number of reference GBS tags that carried SNPs for each SNP caller/alignment combination is given in Table 3. The number of SNP-containing GBS tags common to different references and to different SNP callers is shown in Fig. 2 and Additional file 6: Figure S2. For construction of the genetic maps, the SNPs identified with Unified Genotyper and Haplotype Caller against the references Ref50_98 and Ref70_98 were pooled, yielding a total of 17,245 SNPs distributed over 7307 tags. Consolidation of the SNPs located on the same tag to one consensus SNP per tag reduced the number of SNPs (tags) to 7125. A total of 182 tags was removed because they carried SNPs with conflicting genotypic scores in 20% or more of the progeny. High numbers of conflicting scores across SNPs located on the same GBS tag is likely caused by alignment of both A and B genome reads to the same GBS reference tag.

### Comparison of UGbS-flex and GBS-SNP-CROP pipelines

Using the same dataset and, to the extent possible, same thresholds as reported by Melo and colleagues [13], UGbS-Flex yielded, before filtering, a total of 86,810 SNPs compared to 56,598 reported by Melo and colleagues for GBS-SNP-CROP (Additional file 7: Table S4). After filtering, the total number of SNPs retained from the UGbS-Flex pipeline was 50,139 compared to 21,318 by GBS-SNP-CROP (Additional file 7: Table S4). The SNP number reported for the GBS-SNP-CROP pipeline is when a single accession with the most abundant read number was used in the generation of a reference. When all 48 accessions were used, the total number of filtered SNPs obtained by GBS-SNP-CROP was 14,712. We used all 48 accessions to generate a reference with UGbS-Flex.

### Genetic mapping

**Fig. 3:**
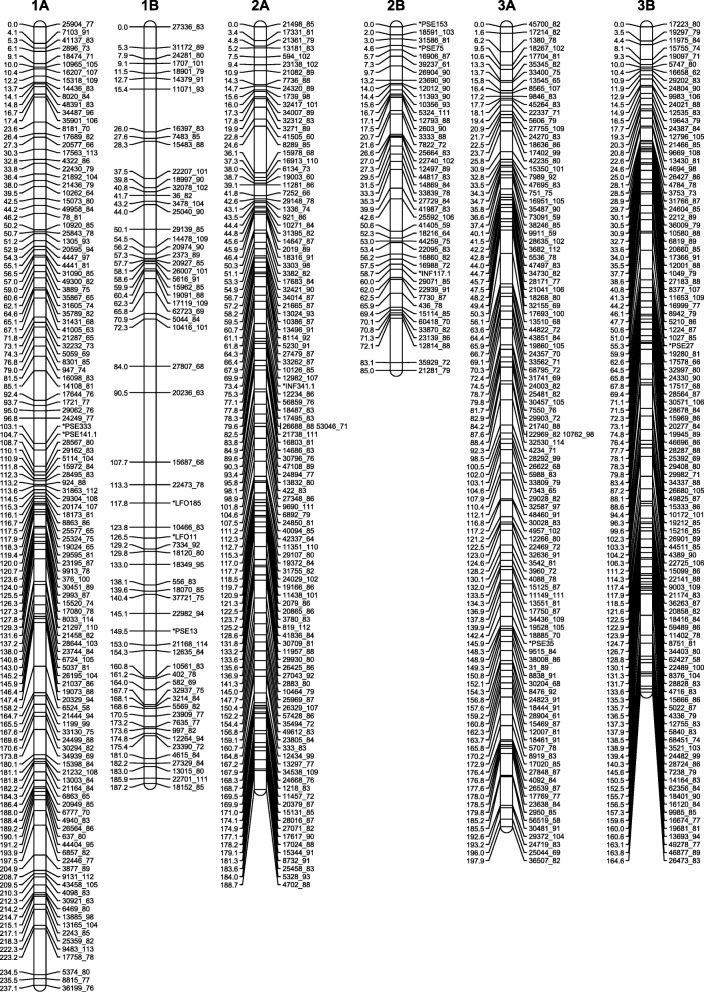
High-density genetic maps of finger millet (homoeologous groups 1, 2 and 3). Marker names are on the right-hand side, centiMorgan (Kosambi) distances on the left-hand side. For readability, only the first marker of each marker bin is represented on the map. Locations of all markers are available from Additional file 9: Table S6

**Fig. 4:**
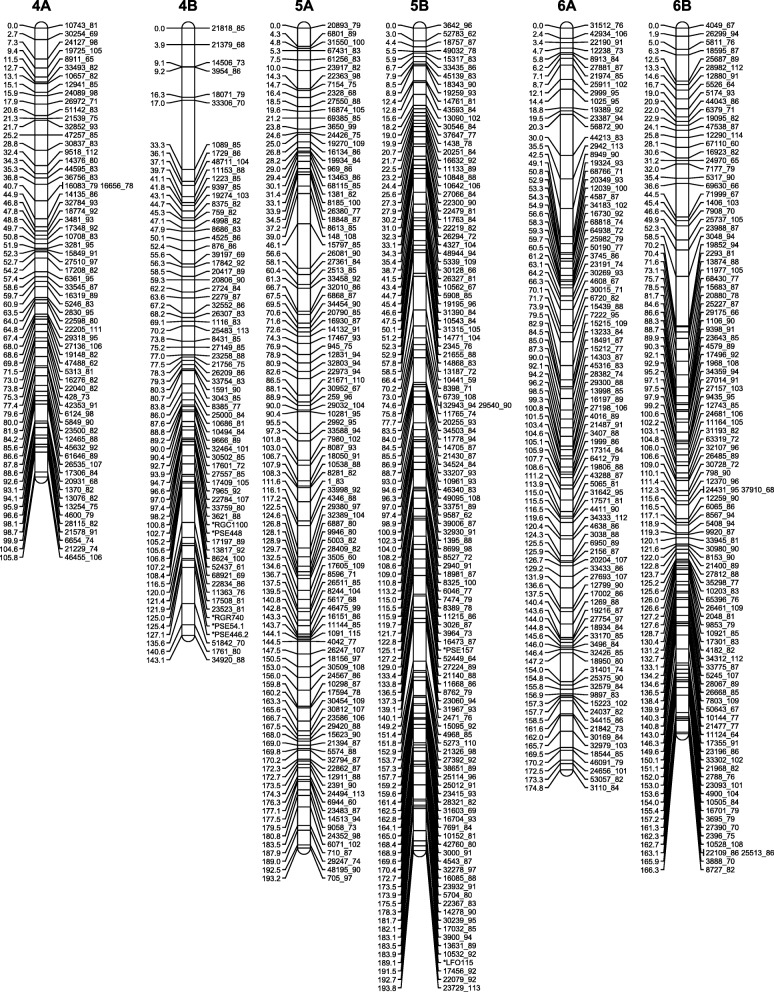
High-density genetic maps of finger millet (homoeologous groups 4, 5 and 6). Marker names are on the right-hand side, centiMorgan (Kosambi) distances on the left-hand side. For readability, only the first marker of each marker bin is represented on the map. Locations of all markers are available from Additional file 9: Table S6

**Fig. 5:**
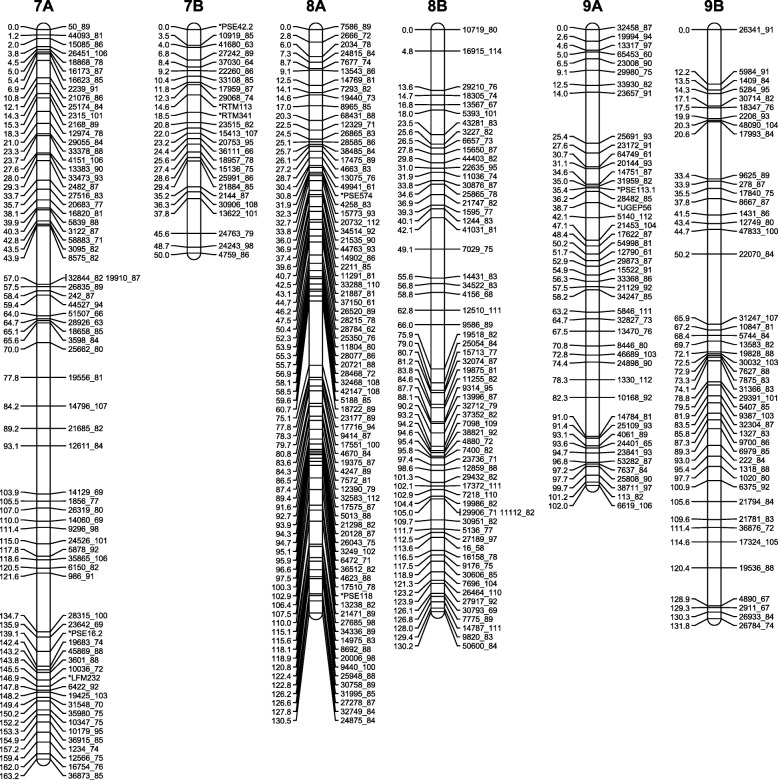
High-density genetic maps of finger millet (homoeologous groups 7, 8 and 9). Marker names are on the right-hand side, centiMorgan (Kosambi) distances on the left-hand side. For readability, only the first marker of each marker bin is represented on the map. Locations of all markers are available from Additional file 9: Table S6

Because some of the distorted markers caused spurious linkages, we initially removed all markers with segregation ratios that deviated from 1:2:1 (A:H:B). We also removed cosegregating markers to reduce marker load during map construction. After the initial map generation, three groups of distorted markers that linked together at high LOD scores and extended the preliminary maps were re-added to the dataset to generate the final maps. The maps consisted of a total of 3772 SNP markers organized in 18 linkage groups with the number of markers per linkage group varying from 39 (51 cM) to 301 (240 cM) (Figs. 3, 4 and 5, Table 4, Additional file 8: Table S5). After integrating the cosegregating markers, the total number of markers mapped was 4453 (Additional file 9: Table S6). The number of recombination bins per chromosome varied from 25 to 120 (Additional file 8: Tables S5 and Additional file 9: Table S6). The sequences of the mapped GBS tags and the SNP positions in these tags are provided in Additional file 9: Table S6.

**Table 4:** Number of markers and map length of each of the nine A and nine B genome linkage groups

		1	2	3	4	5	6	7	8	9
A	Number of markers	301	259	292	162	295	260	204	183	122
	Map length (cM)	239.8	191.5	200.7	108.1	197.2	177.3	163.2	131.6	102.8
B	Number of markers	148	112	295	198	289	251	39	183	179
	Map length (cM)	188.6	85.4	167.1	144	195.5	173.7	50.5	131.4	131.8

### Allocating linkage groups to a and B subgenomes

**Fig. 6:**
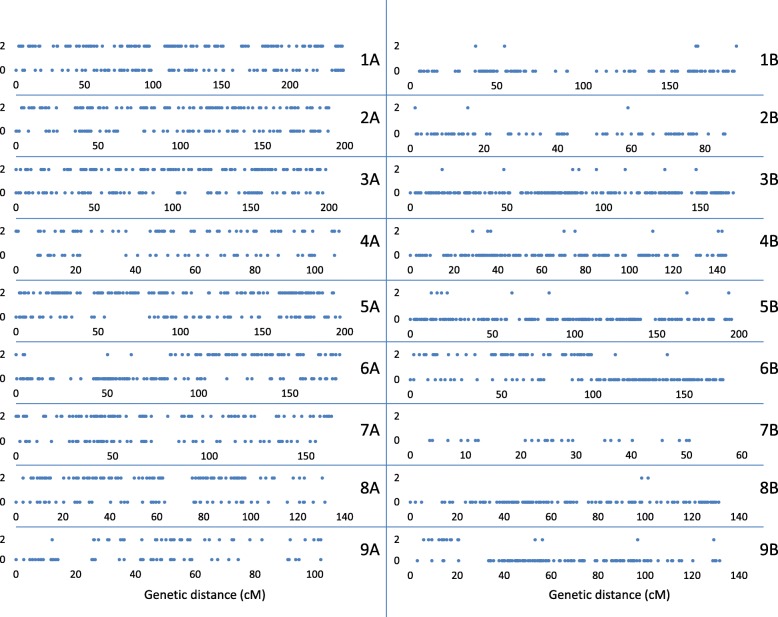
Scatterplots showing presence/absence in *E. indica* (AA genome) of GBS tags mapped in *E. coracana* (AABB genome). Linkage group designations are given on the right-hand side of each graph. GBS tags are ordered by map position (distance in cM). Tags that were present in at least two of the three *E. indica* accessions analyzed were placed at position ‘2’ on the Y-axis. Tags that were absent from all three *E. indica* accessions analyzed were placed at position ‘0’ on the Y-axis. GBS tags located on the B-genome of *E. coracana* are absent from *E. indica*. GBS tags on the A-genome of *E. coracana* are predominantly present (conserved tag) but can be absent from *E. indica* (polymorphism at restriction site, tag not amplified due to a large insertion, or tag not sequenced)

For approximately 13% of the mapped *E. coracana* GBS tags, corresponding GBS reads were identified in all three *E. indica* accessions analyzed. An additional 14% of mapped tags were represented in two of the three analyzed *E. indica* accessions and 18% were present in only a single *E. indica* accession. Excel scatterplots showing the distribution of GBS tags absent from all three *E. indica* accessions and present in at least two of the three *E. indica* accessions in each of the 18 *E. coracana* linkage groups are shown in Fig. 6. Chromosomes within seven of the nine homoeologous groups were unambiguously assigned to the A or B subgenome. A/B translocations were identified for homoeologous groups 6 and 9.

## Discussion

### Optimization of the GBS technology

We tested several modifications to the experimental GBS protocol developed by Elshire et al. [2] and Poland et al. [8]. The aim was to reduce the fragment pool for sequencing and to provide more even read coverage across pooled samples to increase read depth at each locus and SNP representation across samples. A reduction in the number of fragments that will be sequenced can be attained by preventing the addition of Illumina sequencing adapters to a subset of the DNA fragments during the PCR step. This can be achieved using primers with one or more selective bases as applied in Tunable GBS (tGBS®, Data2Bio, ISU Research Park, Iowa) or by cutting PCR-amplifiable fragments with a restriction enzyme. We pioneered the latter approach in finger millet, an inbreeding tetraploid species. DNA of three accessions was either double digested with the enzyme combination *Pst*I/*Msp*I or *Pst*I/*Nde*I, or triple digested by adding *Ape*KI to the *Pst*I/*Msp*I digest. *Pst*I and *Nde*I are 6-bp cutters, *Msp*I is a 4-bp cutter and *Ape*KI is a 5-bp cutter. *Pst*I, *Nde*I and *Ape*KI are sensitive to CNG methylation. No adapters were ligated to sites generated by the third enzyme. While employing a third enzyme did reduce the fragment pool significantly, random ligation between fragments that originated from different parts of the genome during the adapter-ligation step resulted in chimeric fragments. These fragments were sequenced but did not align within or across accessions. Use of a three-enzyme mix therefore did not provide an advantage over a double digest. A third enzyme could likely be employed more effectively after ligation of the adapters, but this would require an additional step in the GBS protocol. The use of *Nde*I, a 6-bp cutter, in the double digest was advantageous for low read numbers (500,000 or less) because a smaller fragment pool allowed more fragments to be sequenced that were common across samples to a depth of at least 8×. For target read numbers in the range of 1 M to 2 M per sample and SNP scoring at a minimum read depth of 8×, we recommend the use of *Pst*I/*Msp*I. The *Pst*I/*Msp*I combination generated a larger fragment pool than *Pst*I/*Nde*I but this fragment pool remained sufficiently small that many fragments that were common across samples were sequenced to the desired 8× depth. Obtaining more than 2 M reads was not cost-effective for fragment pools generated by *Pst*I*/Msp*I (Additional file 3: Table S1).

**Fig. 7:**
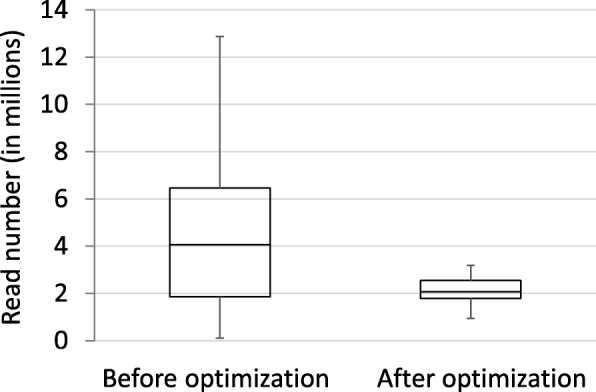
Box-and-whisker plot showing the read number distribution across pooled samples before optimization and after optimization of the GBS protocol

We added two size selection steps to our protocol. Fragments smaller than 300 bp were removed after ligation of the adapters to the DNA fragments using Sera-Mag SpeedBeads. During the PCR step, the duration of the primer elongation step was kept to 15 s which largely limited amplification to fragments shorter than 800 bp. In addition, we incorporated a modification to obtain a more even read number across pooled samples. In the original protocol [2], samples were pooled before the PCR step. Elshire and colleagues initially noted large variation in read numbers between samples which they attributed to inconsistent pipetting by the liquid handling system and rectified by adjustments to the system [2]. We also saw significant variation in read numbers between samples within a pool. While we aimed to obtain two million reads per sample, read numbers varied from 103,000 to close to 13 million in our early GBS experiments (Fig. 7). Variation in pipetting, which was done manually, might have been one contributing factor, but variation between samples in the efficiency of adapter ligation also likely played a role. We therefore PCR amplified samples individually, and measured the DNA concentration on a Qubit fluorometer before pooling equal amounts of DNA. Samples with a DNA concentration less than 5 ng/μl were discarded as they resulted in low read numbers even after adjusting the amount of sample added to the pool. Furthermore, these samples could generally not be rescued by redoing only the PCR step but had to be redone from the start, indicating that the problem lay either in the digestion or adapter-ligation step. Introduction of this modification greatly narrowed the range of read numbers obtained (Fig. 7). While this approach increased the cost of sample preparation, it allowed ‘bad samples’ to be identified pre-sequencing and, as such, represented a cost-saving at the sequencing end.

### Generation of a GBS reference

We wanted a GBS protocol and analysis pipeline that would provide several thousand robust SNP markers in a variety of species, irrespective of the breeding system, ploidy level, polymorphism level and availability of a whole-genome sequence. To maximize the chances of finding SNP variation in species with unknown or low levels of variation, we opted to sequence the reduced representation libraries from both ends (2 × 150 bp on a NextSeq). Furthermore, the longer paired-end reads were more likely to identify orthologous sequences in related species, and availability of comparative information is key to the study of orphan crops with few genetic and genomic resources. While a few pipelines (e.g. TASSEL-GBS [10], STACKS [12]) were available when we initiated our analyses that could build a genomic reference from the GBS tags, none could deal with paired-end reads or had been developed for data from polyploid species*.* These shortcomings were recently addressed with the publication of the GBS-SNP-CROP pipeline [13]. We present an alternative pipeline, UGbS-Flex, that can generate a GBS reference from paired-end data from inbreeding diploids as well as outcrossing allopolyploids. This intuitive and flexible pipeline consists of publicly available software packages and in-house perl and python scripts to cover data analysis from read processing to SNP scoring (Fig. 1). Each UGbS-Flex module can be run independently.

Much of the UGbS-Flex pipeline development was done with GBS sequencing data generated in an F_2_ population of finger millet. However, we have successfully used the UGbS-Flex pipeline for the analysis of GBS data generated in diversity panels in finger millet and foxtail lily, and in mapping populations in seashore paspalum, an outcrossing diploid, and switchgrass, an outcrossing tetraploid. To facilitate working with paired-end data, we merged overlapping sequences, and considered non-overlapping paired-end reads as contiguous sequences. We then grouped reads by similarity within samples using the ‘ustacks’ module from the STACKs package [12] and across samples using either ‘cstacks’ or the novel ‘ASustacks’ approach. The latter applies ‘ustacks’ to a file that comprises consensus sequences for per sample read clusters from all samples. Because the ‘ASustacks’ approach is more efficient in terms of computing resources and numbers of identified GBS reference tags, it has been integrated in the UGbS-Flex pipeline for analysis of reduced representation sequencing data generated using methylation-sensitive restriction enzymes and hence enriched for low copy sequences.

Optimal ‘ustacks’ and ‘cstacks/‘ASustacks’ parameters are dependent on the breeding system, ploidy, and polymorphism level of the species under investigation. For example, in an inbreeding species, most loci are homozygous, and stacks within an accession should be built with reads containing no or only a single mismatch. Use of very stringent conditions in the stack building within inbreeding individuals will promote separation of paralogs and, in polyploids, of homoeologous loci. In outcrossing polyploid species, however, stacks within accessions need to be generated that comprise homo-alleles but not homoeo-alleles. Finding the optimal parameters to achieve this can be challenging, in particular in highly polymorphic polyploids, and needs to be done empirically. Similar considerations need to be taken into account when generating stacks across accessions. For finger millet, we allowed a 1 bp mismatch both for clustering within samples and across samples. Polymorphism levels in finger millet are relatively low, and because we were working with an F_2_ population, allelic variation within and between samples was similar. The ‘cstacks’/‘ASustacks’ outputs were filtered to retain only those consensus sequences that were present in a specified percentage of the samples, and these sequences were used as a reference. This step limits downstream identification of SNPs with large amounts of missing data.

### SNP calling

**Fig. 8:**
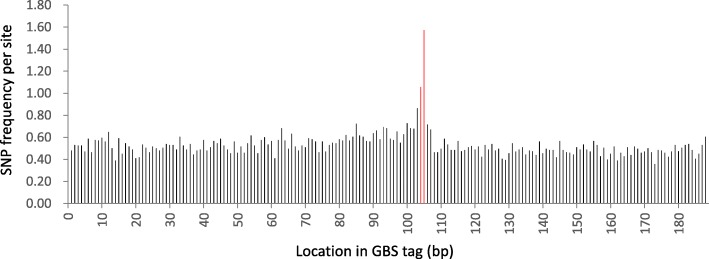
SNP distribution across *Pst*I/*Msp*I GBS tags for which forward and reverse reads were joined artificially during the GBS reference generation in three finger millet accessions (KNE 796, MD-20 and Okhale-1). Red bars flank the junction between forward and reverse reads

Quality-trimmed reads were aligned to the GBS reference using Bowtie 2 [27]. SNPs were called using GATK [28]. The fact that the GBS reference consisted of paired-end reads that were artificially joined but were physically separated in the genome did not affect the bowtie alignment nor the SNP calling. The only exception was when a deletion was present in an allele relative to the allele used in the GBS reference. Because alleles are sequenced to a fixed length on Illumina platforms (e.g. 150 bp), the presence of a, for example, 2-bp deletion polymorphism in the region sequenced practically means that sequence information will be generated for an additional 2 bp at the 3’end of reads for alleles with the deletion compared to alleles without the deletion. If the corresponding GBS reference tag lacks the deletion, there will be an alignment gap at the location of the deletion and the ‘extra’ 2 bps sequenced will extend beyond the junction point where the forward and reverse reads were artificially joined in the GBS reference. This leads to SNP artifacts (Additional file 10: Figure S3). Because adjacent SNPs were removed as part of our SNP filtering protocol, only spurious SNPs caused by single base pair deletions remained in our dataset. The SNP frequency immediately flanking the junction between the forward and reverse reads was two-to three-fold higher than the SNP frequency across the rest of the read (Fig. 8). Discarding the SNPs at these two positions reduced overall SNP numbers by approximately 2.5%.

We tested both GATK’s Haplotype Caller and Unified Genotyper for calling SNPs. Unified Genotyper identified some 17% more SNPs than Haplotype Caller (Table 3). On average, 25% of the SNPs identified with Unified Genotyper were not identified by Haplotype Caller (Fig. 2, Additional file 6: Figure S2). Conversely, some 12% of SNPs identified with Haplotype Caller were not identified by Unified Genotyper. These comparisons were made on filtered data sets, and hence concerned robust SNPs. The percentage of SNPs uniquely identified by Unified Genotyper and Haplotype Caller that were incorporated in the genetic maps and hence were validated, was very similar (41.3% *vs* 40.1% for Ref50_98 and 47.5% *vs* 40.8% for Ref70_98). This indicates that, at least in our dataset, the high sensitivity/aggressiveness of Unified Genotyper in calling SNPs did not increase the false positive rate. For comparison, the percentage of SNPs identified by both SNP callers that were incorporated in the genetic maps was 56% for Ref50_98 and 55.4% for Ref70_50. Freebayes [29], a haplotype-based SNP caller, also yielded a lower number of SNPs than Unified Genotyper when tested on the parents of the mapping population. Although we did not validate the SNPs identified by Freebayes, the number of SNPs identified in common with Unified Genotyper and the percentage of common SNPs mapped suggest that the lower SNP detection rate in Freebayes would have resulted in a lower percentage of mappable polymorphisms.

The threshold number of samples in which each representative GBS tag had to be present in order to be included in the GBS reference also greatly affected the number of identified SNPs, although the trend was opposite to what we expected (Table 3). To our surprise, we identified fewer SNPs, after filtering, when a representative tag was selected to be present in at least 50% of the samples (Ref50) compared to at least 70% (Ref70), despite the fact that more reference tags were present in the Ref50 compared to the Ref70 reference. We hypothesized that, although the Ref70 reference carried fewer tags, more loci were represented by only a single allele. If alleles from the same locus (e.g. Parent 1 allele and Parent 2 allele) formed separate stacks during the clustering performed by ‘ustacks’, both would be included in the GBS reference. Reads from the same locus in heterozygous individuals would align to either the allele 1 GBS reference tag or the allele 2 GBS reference tag during the bowtie alignment, and be scored as two homozygous loci in GATK. To test this hypothesis, we removed tags that were potentially allelic to another tag. When a GBS tag had more than 98% homology to another tag following a blast-all-against-all analysis, only one of the tags was included in the GBS reference. Removing allelic tags from Ref50 (referred to as Ref50_98) resulted in the calling of 136% new SNPs, while less than 2% of SNPs were lost (Additional file 6: Figure S2). As expected, SNP gain was significantly smaller (15%) when allelic tags were removed from Ref70 (referred to as Ref70_98), but still greatly outpaced SNP loss (1%) (Additional file 6: Figure S2). SNP discovery now followed the expected trend with approximately 24% higher SNP numbers being identified with Ref50_98 compared to Ref70_98 (Fig. 2). Interestingly, only 75% of the SNPs identified with Ref70_98 were detected with Ref50_98. The percentage of SNPs uniquely found by only a single reference was 25 and 39% for Ref70_98 and Ref50_98, respectively. This suggest that a substantially improved reference could be obtained and SNP calling maximized by combining the tags in both GBS references.

The UGbS-Flex pipeline compares favorably to GBS-SNP-CROP [13], which can also use paired-end reads as input. Using the same filtering criteria, the number of SNPs identified by UGbS-Flex was more than 3-fold that identified by GBS-SNP-CROP when the reference in both pipelines was generated with all 48 accessions. Interestingly, more heterozygotes were identified by GBS-SNP-CROP than UGbS-Flex. While a larger percentage of the SNPs identified by GBS-SNP-CROP than UGbS-Flex (99.9% *vs* 73.7%) had read depths ≥20, it seems unlikely that the read depth explains the difference in the calling of homozygotes *vs* heterozygotes between the two pipelines because of the stringent criteria used in SNP calling. As per Melo and colleagues [13], to score a SNP locus as homozygote required a minimum read depth of 11 when the secondary allele count was zero, and a minimum read depth of 48 when the secondary allele count was one. Because kiwiberry is a tetraploid, it is likely that some of the heterozygote SNPs are variants between homoeo-alleles rather than homo-alleles. The parameters used to cluster reads may have separated homoeologs from homologs at a higher frequency in UGbS-Flex than in GBS-SNP-CROP.

### Construction of a high-density genetic map

We had previously constructed a 332-loci genetic map in an F_2_ population generated from a cross between the wild *E. coracana* subsp. *africana* acc. MD-20 and the cultivated *E. coracana* subsp. *coracana* acc. Okhale-1 using restriction fragment length polymorphism (RFLP), simple sequence repeat (SSR) and expressed sequenced tag (EST) markers [15, 16]. The same population was used here to generate a high-density genetic map. The decision to use a three-enzyme combination (*Pst*I/*Msp*I plus *Ape*KI) for GBS of the mapping population predated data availability on the relative efficiency of different enzyme combinations in generating polymorphic markers. The choice of enzyme combination does not affect map quality, only marker number. Because DNA from individual F_2_ plants was no longer available, we used DNA extracted from bulked F_2:3_ families for mapping. The drawback of this was that the mapping data was not quite as clean as when genotyping actual F_2_ plants, especially in heterozygous regions that underwent recombination or displayed segregation distortion. Mapping programs such as MSTmap [21] and Lep-Map [22] are based on the traveling salesman principle (TSP) and can very quickly generate maps with large numbers of markers. However, because marker ordering relies on two-point linkage information, TSP mapping programs are more affected by missing data and provide less robust genetic maps than programs such as MAPMAKER [17] that use multipoint analyses [30] (Additional file 11: Figure S4). Furthermore, C (ambiguous B or H) and D (ambiguous A or H) values could be incorporated in MAPMAKER, but had to be converted to missing data points in MSTmap. We therefore used a hybrid approach in which we identified linkage groups and did the initial marker ordering with MSTmap. Using the MSTmap marker orders, we generated maps in MAPMAKER with the option ‘error detection on’ to identify markers with high levels of genotypic errors. These markers were removed from further analyses. We then used the MSTmap marker orders to select overlapping marker groups for fine-scale ordering using three/multipoint analyses in MAPMAKER. The version of MAPMAKER employed (available from http://research.franklin.uga.edu/devoslab/) had been modified to run efficiently in a Windows Command Prompt Environment and to handle larger numbers of markers than the original MAPMAKER version [18]. Despite the modifications, marker ordering was still limited to groups of approximately 100 markers due to inherent software limitations. The ‘try’ and ‘ripple’ commands in MAPMAKER were semi-automated with in-house developed python scripts. Because the overlapping segments were selected based on the initial marker orders defined by MSTmap, incorrect placement of one or more markers in the MST map could affect the final MAPMAKER-generated map. Recombination events in the final maps were therefore scrutinized manually and, if necessary, potentially problematic map regions were reanalyzed. Two blocks of markers, one on linkage group 2A and one on 2B, were removed because they were flanked on either side by a large number of recombination events. Following the manual assignment of markers to recombination bins and addition of the cosegregating markers, we obtained a robust map consisting of 4453 SNP markers organized in 18 linkage groups (Figs. 3, 4 and 5; Additional file 9: Table S6).

### The finger millet genetic map and its characteristics

**Fig. 9:**
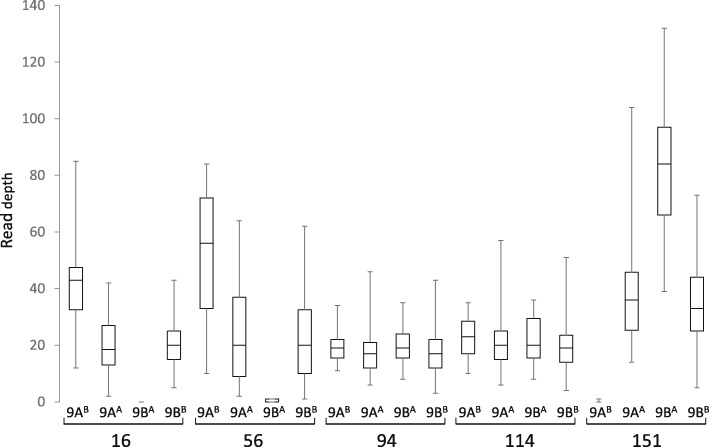
Box-and-whisker plots showing the read depth distribution at SNP positions along the length of the translocated (9A^B^ and 9B^A^) and non-translocated regions (9A^A^ and 9B^B^) in chromosomes 9A and 9B of five selected progeny. Progenies 16 and 56 carry a chromosome complement in which the 9B^A^ region is absent and two copies of the 9A^B^ region are present. Progeny 94 and 114 carry complete A and B genomes. Progeny 151 carries a chromosome complement in which the 9A^B^ region is absent and two copies of the 9B^A^ region are present

The 18 *E. coracana* linkage groups were labeled 1 to 9 with the suffix A or B to designate whether they originated from the A or B subgenome (Figs. 3, 4 and 5; Additional file 9: Table S6). Linkage group designations were the same as in Dida et al. [15] as determined by the incorporation of a subset of the markers from the map generated by Dida and colleagues into the high density GBS map. The assignment of each linkage group to a subgenome had previously been achieved by identifying a small number of RFLP markers for each linkage group that were conserved in size between *E. indica*, the A genome progenitor of *E. coracana*, and the presumed A genome of *E. coracana* [15]. The GBS data generated in *E. indica* provided us with an opportunity to scan the entire length of each linkage group for the presence of A-genome markers. A-genome linkage groups were expected to carry *E. indica* GBS tags along their entire length, while B-genome linkage groups should be devoid of such tags. We observed the expected pattern in seven of the nine homoeologous linkage groups (Fig. 6). Interestingly, in homoeologous groups 6 and 9, the presence/absence pattern of *E. indica* GBS tags indicated the presence of a reciprocal translocation between homoeologous chromosomes. Visual examination of the group 9 recombination data (Additional file 8: Table S5) showed a number of progeny that carried recombination events in both the A and B homoeologs at the putative translocation breakpoint leading to cross-shaped maps which had been split at high LOD scores during our mapping analysis. This suggests that the group 9 translocation was present in only one of the two mapping parents. Chromosomes that are heterozygous for a translocation undergo pairing in a cross-type configuration. Depending on how the chromosomes segregate (and assuming no cross-overs take place), progeny can either carry full copies of the A and B genome chromosomes or a chromosome complement that carries deletions/duplications for regions of the translocated chromosome. One progeny (progeny 151 in Additional file 8: Table S5) indeed lacked all A-genome markers located within the translocated region and five progeny (16, 56, 125, 131, 148) lacked the B-genome markers, indicating that these regions were deleted in those progeny. Further analysis showed that read numbers were approximately double in the corresponding B-genome region in progeny 151 and in the corresponding A-genome region in progenies 16, 56, 125, 131 and 148, indicating that the absence of a region was compensated for by an extra copy of the homoeologous region in those progeny (Fig. 9). While deletion of a chromosomal region would likely be deleterious in a diploid species, the presence of homoeologous chromosomes in an allopolyploid largely buffers against the negative effects caused by chromosomal deletions. Progeny with apparent recombination events in homoeologous A and B chromosomes, or with a deleted region were not identified for chromosomes 6A and 6B. It is possible that the 6A/6B rearrangement occurred early in polyploid evolution and is present in both parents. Identification of a heterozygous translocation through the comparison with *E. indica* depends on whether the cross-shaped maps were split into two non-translocated chromosomes or into two translocated chromosomes. We therefore scrutinized the other homoeologous groups for cross-shaped linkages and deletions, and uncovered an interstitial rearrangement in homoeologous group 2. The two interstitial marker blocks that we removed during the map construction, because they were flanked on either side by a large number of recombination events, were regions that had undergone an interstitial translocation in one of the parents. At this point, we do not know whether the 9A/9B and 2A/2B rearrangements occurred in the cultivated or wild parent. In allopolyploids, pairing is typically controlled genetically and limited to homologous chromosomes [31, 32]. Removal of the pairing control locus can lead to chromosomal rearrangements, including homoeologous translocations [33, 34]. Finger millet has been reported to display disomic inheritance [35, 36] but the mechanism of pairing control is not known. An analysis of the pairing behavior is needed to determine whether homoeologous pairing control is suppressed in either of the parents.

## Conclusions

We provide a detailed analysis of the effect of parameter and module changes in the GBS methodology, reference generation and SNP calling that were aimed at maximizing the discovery of high confidence SNPs with minimal missing data. The newly developed UGbS-Flex pipeline provides a useful addition to the currently available tools for GBS data analysis, in particular for genotyping of heterozygous and polyploid individuals from paired-end sequencing reads in the absence of a whole-genome reference. The UGbS-Flex pipeline was applied here to identify high confidence SNPs that were subsequently used to generate the first high-density genetic map of finger millet, *Eleusine coracana*. Map robustness was achieved by applying multipoint marker ordering. We demonstrated that cross-species application of GBS is feasible between closely related species and, when applied between an allopolyploid and one of the diploid genome donors, can be used to identify the subgenome origin of linkage groups and the occurrence of translocations between homoeologous chromosomes. Finger millet, an inbreeding allopolyploid species with few genomic resources was used here as a case study, but applicability of the UGbS-Flex pipeline to other species, including the outcrossing tetraploid switchgrass has been successfully achieved.

## Additional files

Additional file 1: **Data S1.** System requirements and UGbS-Flex commands for GBS reference generation, SNP calling and further SNP processing. (PDF 165 kb)

Additional file 2: **Figure S1.** The ‘Bcraw’ folder comprises the raw sequencing files for individual samples. After trimming, the trimmed sequence files are placed in the ‘BCpc’ folder. Using files in the ‘BCpc’ folder as input, all files with equal-length reads are placed in ‘BCfin’ folder. The ‘ASU’ folder holds the ASU method results for all files present in the ‘BCfin’ folder. The ASU results are used to generate a reference; the filtered reference is placed in the ‘Ref’ folder. The trimmed sequences in the ‘BCpc’ folder are aligned (with Bowtie) against the reference files in the ‘Ref’ folder; alignments are used for SNP calling (using GATK); all results are stored in the ‘SNP’ folder. The ‘Process’ number corresponds to the step number in Additional file 1: Data S1. (PPTX 52 kb)

Additional file 3: **Table S1A.** Summary statistics obtained for each of the three enzyme combinations for subsets of reads; Entries are grouped by read number. **Table S1B.** Summary statistics obtained for each of the three enzyme combinations for subsets of reads; Entries are grouped by enzyme combination. (XLSX 29 kb)

Additional file 4: **Table S2.** Average read depth (across three accessions tested) of GBS reference tags common to all three accessions in the *Pst*I/*Nde*I fragment pool. (DOCX 13 kb)

Additional file 5: **Table S3.** Number and percentage of *Ape*KI sites present in *Pst*I/*Msp*I and *Pst*I/*Msp*I + *Ape*KI digests. (DOCX 12 kb)

Additional file 6: **Figure S2.** Comparison of the number of SNPs identified using different SNP callers (UG = Unified Genotyper; HC=Haplotype Caller) and different GBS references (Ref50: tags present in ≥50% of the samples; Ref70: tags present in ≥70% of the samples; Ref50_98: tags present in ≥50% of the samples and only 1 tag retained for tags with ≥98% homology; Ref70_98: tags present in ≥70% of the samples and only 1 tag retained for tags with ≥98% homology. (PPTX 7261 kb)

Additional file 7: **Table S4.** Comparison of SNPs identified by UGbS-Flex and GBS-SNP-CROP. (XLSX 9 kb)

Additional file 8: **Table S5.** Genotypic data for the MD-20 x Okhale-1 population. (XLSX 2444 kb)

Additional file 9: **Table S6.** Genetic maps comprising all markers. (XLSX 717 kb)

Additional file 10: **Figure S3.** Effect of the presence of a deletion in a sample relative to the GBS reference allele. A gapped alignment is formed and the 3′ end extends beyond the junction of the forward and reverse reads in the GBS reference resulting in the calling of a SNP at that position. The Integrative Genomics Viewer (Robinson et al. 2011, Nature Biotechnology 29: 24–26; Thorvaldsdóttir et al. 2013, Briefings in Bioinformatics 14: 178–192) was used for visualization. (PPTX 75 kb)

Additional file 11: **Figure S4.** Comparison of genetic maps generated using MSTmap (left-hand side) and MAPMAKER (right-hand side). Nearly 65% of markers were reordered in MAPMAKER compared to MSTmap maps. The markers that occupied a different relative position in the two maps are connected by a line. (PPTX 170 kb)
