# Supplementary material for: UGbS-Flex, a novel bioinformatics pipeline for imputation-free SNP discovery in polyploids without a reference genome: finger millet as a case study
Source: BMC Plant Biol. 2018 Jun 15;18:117. doi: 10.1186/s12870-018-1316-3 (PMC6003085; doi:10.1186/s12870-018-1316-3)
Supplement: Supplementary file 1 — Data S1. System requirements and UGbS-Flex commands for GBS reference generation, SNP calling and further SNP processing. (PDF 165 kb) [file 12870_2018_1316_MOESM1_ESM.pdf]

Supplementary Data S1

S1. System requirements

OS: Linux or UNIX for S2, no specific requirement for S3

Software packages: Fastqc (v 0.11.4), Stacks (v .1.44), FastX (v.0.0.14), Flash (v.1.2.11), bowtie2 (v.2.2.9), samtools (v.1.3.1), picard (v.2.4.1), GATK (v3.4.0), perl (v.5.20.1), Python (v.3.5.1 or higher)

Make sure the Python version is equal to or higher than v 3.0; codes are not compatible with any Python ver 2.x.x. Scripts work under Linux/UNIX/Windows/MacOS.

S2. Generate reference from raw reads

To generate a reference from raw reads:

1) Check sequencing quality of raw reads with '*fastqc*' [1], and output results to a new directory "Fastqc_result"

   The script:
   fastqc/0.11.4/fastqc raw_reads_lane1_read1.fastq -o Fastqc_result

2) Split the raw reads according to their barcode using '*process_radtags*' from Stacks [2].
   For reads from each sequencing lane, make a separate barcode file for each length of barcode, run scripts below for each barcode file, and put the outputs in separate folders for each lane (like "lane1"). Check Stacks documentation for details and parameters.

   The script:
   stacks/1.44/bin/process_radtags -1 raw_reads_lane1_read1.fastq -2 raw_reads_lane1_read2.fastq –b barcode.txt –o lane1 --renz_1 pstI –r --adapter_1 CACGACGCTCTTCCGATCT --adapter_2 CGAGATCGGAAGAGCGG --adapter_mm 1

3) If applicable, merge reads from the same sample sequenced on multiple sequencing lanes, and output the result to a new directory "BCraw". Do this separately for read 1 and read 2. Make sure the files are merged in the same order for read 1 and read 2.

   The script:
   cat lane1/sample.1.fq lane2/sample.1.fq > BCraw/sample1.1.fq
   cat lane1/sample.2.fq lane2/sample.2.fq > BCraw/sample1.2.fq

4) Using step 3 output as input, trim reads to remove restriction sites and low quality bases using '*fastx_trimmer*' (http://hannonlab.cshl.edu/fastx_toolkit/). Put output in a new directory "BCpc"

   The script:
   fastx/0.0.14/bin/fastx_trimmer –Q 33 –f 6 -l 135 –i sample1.1.fq -o BCpc/sample1.1.fq
   fastx/0.0.14/bin/fastx_trimmer –Q 33 –f 4 -l 145 –i sample1.2.fq -o BCpc/sample1.2.fq

Use –f 6 for *Pst*I, -f 4 for *Msp*I, -f 5 for *Nde*I, -l 135 for the forward reads, -l 145 for the reverse reads (these values are for a raw read length of 150 bp; -l is the read length remaining after removal of barcodes and five lower quality bases at the 3' end of reads).

Example (using demo files):

$ mkdir BCpc
$ fastx_trimmer –Q 33 –f 6 -l 135 –i 1M_PM-KNE10.1.fq -o BCpc/ 1M_PM-KNE10.1.fq
$ fastx_trimmer –Q 33 –f 4 -l 145 –i 1M_PM-KNE10.2.fq -o BCpc/ 1M_PM-KNE10.2.fq

Do this for each of the three demo samples.

5) In the "BCpc" directory, merge overlapping paired reads using '*flash*' [3]. Do this for each sample.

The script:
flash/1.2.11/flash sample1.1.fq sample1.2.fq

Flash produces three files of interest: *out.extendedFrags.fastq* which contains the merged overlapping paired-end fragments, *out.notCombined_1.fastq* which contains the non-overlapping forward reads and *out.notCombined_2.fastq* which contains the corresponding non-overlapping reverse reads.

Rename these three files as follows:
mv out.extendedFrags.fastq sample1.Comb.fq

mv out.notCombined_1.fastq sample1.Solo.1.fq

mv out.notCombined_2.fastq sample1.Solo.2.fq

Example (using demo files):

$ flash 1M_PM-KNE10.1.fq 1M_PM-KNE10.2.fq
$ mv out.extendedFrags.fastq 1M_PM-KNE10.Comb.fq
$ mv out.notCombined_1.fastq 1M_PM-KNE10.Solo.1.fq
$ mv out.notCombined_2.fastq 1M_PM-KNE10.Solo.2.fq

Do this for each of the three demo samples.

6) Put python *script EL.1.1.py* (EL for 'equal length') in the same folder as the flash output files. Python version must be 3.0 or higher. Run *EL.1.1.py* by double clicking *EL.1.1.py* in Windows/MacOS or typing "*python3 EL.1.1.py*" in Linux.

This script will (1) remove reads from the *Solo.1.fq* and *Solo.2.fq* files that are shorter than the expected (trimmed) size (if a forward read is removed, the corresponding reverse read is also removed, and *vice versa*), (2) reverse complement the reverse reads in the *Solo.2.fq* file and artificially join them to the 3' end of forward reads in the *Solo.1.fq* file, and (3) extend merged

overlapping reads at the 3' end with 'As' to make them the same length as the joined non-overlapping reads.

The script will ask for the following inputs: Comb file full name (*sample1.Comb.fq*), Solo 1 file name (*sample1.Solo.1.fq*), Solo 2 file name(*sample1.Solo.2.fq*), read length for Solo 1 reads (any solo 1 reads shorter than this number will be removed), read length for Solo 2 reads (any solo 2 reads shorter than this number will be removed), and output file name (for example: *sample.fq*). The *EL1.1.py* script will generate two output files, *sample.fq* for ustacks input, and *dump_sample.fq* which contains all removed reads.

Example (using demo files):

$ python EL.1.1.py
When asked to 'Enter comb reads file full name:', enter: *1M_PM-KNE10.Comb.fq*
When asked to 'Enter Solo 1 reads file full name:', enter: *1M_PM-KNE10.Solo.1.fq*
When asked to 'Enter Solo 2 reads file full name:', enter: *1M_PM-KNE10.Solo.2.fq*
When asked to 'enter the read length standard for Solo 1:', enter: 104
When asked to 'enter the read length standard for Solo 2:', enter: 84
When asked to 'enter output file full name:', enter: *1M_PM-KNE10.fq*

Do this for each of the three demo samples.

7) Use '*ustacks*' to cluster reads within each sample and generate a consensus sequence for each cluster; place the output file into a new directory "ASU". Check Stacks documentation for details and explanation of parameters.

The script:
stacks/1.44/bin/ustacks -t fastq -f BCfin/sample1.fq -o ASU/ -I 1 -m 2 -M 1 -N 1 -H --max_locus_stacks 2

The parameters used in the stack formation will vary with the breeding system and polymorphism level of the species under investigation.

Example (using demo files):

$ mkdir temp
*$ mv *.Comb. * *.Solo.* temp*
$ mkdir BCfin
*$ mv *.fq ../BCfin*
$ cd ../BCfin
$ mkdir ASU
$ ustacks -t fastq -f 1M_PM-KNE10.fq -o ../ASU -I 1 -m 2 -M 1 -N 1 -H --max_locus_stacks 2

Do this for each of the three demo samples.

8) Use ‘*cstacks*’ to cluster consensus sequences (generated by ‘*ustacks*’) across samples and output to a new directory “Cstacks”. Check Stacks documentation for details and explanation of parameters.

The script:
Stacks/1.44/bin/cstacks -b 1 -s sample1 -s sample2 -s sample3 -o Cstacks/ -n 1 -p 8 --report_mmatches.

For large number of samples (more than 100), we suggest to set -p as high as possible (maximum to CPU threads of computer) to decrease calculation duration. ‘cstacks’ on a large number of samples is computationally very intensive. ‘ASustacks’ (see below) can be run as an alternative (recommended). As for *‘ustacks*’, parameters will need to be adjusted based on polymorphism level of the species under investigation.

9) Filter cstacks output and generate reference sequence.
In ‘Cstacks’ folder, use *FCT.pl* to filter the tags generated by ‘*cstacks*’; the minimum (will determine the number of missing data allowed) and maximum number of accession (typically all accessions) that have to be included in a consensus stack is given by the first and second numbers; only consensus tags will be selected that consist of maximum one ‘*ustack*’ tag per accession.

The script:
perl FCT.pl batch_1.catalog.tags.tsv 2 3

Then rename the output to reference.
mv cstacks_output_unique_consensus_multi_fasta ref.fa

To use the ‘*ASustacks*’ module instead of ‘*cstsacks*’ for the generation of stacks across accessions (recommended), replace steps 8 and 9 with the steps listed below:

a. Copy the python script ‘*ASustacks.py*’ to the “ASU” folder, and run ‘*ASustacks.py*’ for each sample:

python3 ASustacks.py

The script will ask for the file input; enter the full name of the *tags.tsv* file for a sample (for example, sample1.tags.tsv). The script will output an artificial .fastq file *sample1_u.fq*.

Example (using demo files):
$ cd ../ASU
$ python3 ASustacks.py
When asked to 'Enter the Ustacks output tags.tsv file:', enter: 1M_PM-KNE10.tags.tsv
When asked to 'Enter the output fastq filename:', enter: 1M_PM-KNE10.fq

Do this for each of the three demo samples.

b. Merge the artificial .fastq files from all samples into a single file. Use the script below:
cat sample1_u.fq sample2_u.fq sample3_u.fq > all_u.fastq

Run '*ustacks'* on the file *all_u.fq* using the script:
stacks/1.44/bin/ustacks -t fastq -f all_u.fq -I 1 -m 1 -M 1 -N 1 -H --max_locus_stacks 2

Example (using demo files):

$ cat 1M_PM-KNE10.fq 1M_PM-MD20.fq 1M_PM-OK.fq > all_u.fastq
$ ustacks -t fastq -f all_u.fq -I 1 -m 1 -M 1 -N 1 -H --max_locus_stacks 2

c. Generate a reference from file *all_u.tags.tsv*. Copy the python script '*ASustacks_ref.py*' to the same folder as *all_u.tags.tsv*. Run the script:

python3 ASustacks_ref.py

The script will ask the following questions upon running: 1) full name of *all_u.tags.tsv*; 2) the number of samples required for using a tag as reference; 3) the output file name. For example, if the output of step c has the file name *all_u.tags.tsv*, the total sample number is 100, and a tag must exist in at least 20 samples to be included in the reference, then enter '*all_u.tags.tsv*' for question 1, and '*20*' for question 2.
The script will output a fasta file; continue to step 10.

Example (using demo files):

$ python3 ASustacks_ref.py
When asked to 'Enter Ustacks output tags.tsv file name:', enter: *all_u.tags.tsv*
When asked to 'Enter output reference fasta file name:', enter: ref.fa
When asked to 'Enter the threshold number for reference', enter: *1*
When asked to 'Enter output reference fasta file name:', enter: *ref.fa*

10) Filter reference to remove tags with 98% similarity.
In the same folder that contains *ref.fa*, run the scripts below:

ncbiblast+/2.2.29/bin/makeblastdb -in ref.fa -dbtype nucl -title refdb -parse_seqids -out refdb refdb.log
ncbiblast+/2.2.29/bin/blastn -db refdb -query ref.fa -evalue 1e-5 -outfmt 6 -max_target_seqs 2 -out ref.blast

Example (using demo files):

ncbiblast+/2.2.29/bin/makeblastdb -in ref.fa -dbtype nucl -title refdb -parse_seqids -out refdb refdb.log

ncbiblast+/2.2.29/bin/blastn -db refdb -query ref.fa -evalue 1e-5 -outfmt 6 -max_target_seqs 2 -out ref.blast

Then, copy Ref_98.py into the same folder and run the script:

python3 Ref_98.py

The script asks for the reference sequence and reference blast result as inputs; enter the full name of *ref.fa* and *ref.blast*. The script's output is the filtered reference *ref_98.fa*. This is the reference to which the trimmed reads will be aligned.

> Example (using demo files):
>
> *$ python3 ASustacks_ref.py*
> When asked to 'Enter the reference fasta file:', enter: *ref.fa*
> When asked to 'Enter the reference blast file:', enter: *ref.blast*

S3. Align GBS reads to reference and SNP calling

11) Prepare a reference index for bowtie2 [4] and GATK [5]
Copy *ref_98.fa* to the "BCpc" folder, and run the scripts below:

bowtie2/2.2.9/bin/bowtie2-build ref_98.fa ref_98
java -jar picard/2.4.1/CreateSequenceDictionary.jar -R=ref_98.fa O=ref_98.dict
samtools faidx ref_98.fa

> Example (using demo files):
>
> *$ bowtie2-build ref_98.fa ref_98*
> *$ java -jar picard.jar CreateSequenceDictionary.jar R=ref_98.fa O=ref_98.dict*
> *$ samtools faidx ref_98.fa*

12) Align reads to the reference and process output for GATK
In "BCpc" folder, run the scripts below for each sample.

bowtie2/bin/bowtie2 -x ref_98 -1 sample1.1.fq -2 sample1.2.fq -S sample1.sam
samtools/1.3.1/samtools view -bS sample1.sam > sample1.bam
java -jar picard/2.4.1/ValidateSamFile.jar INPUT= sample1.bam
java -jar picard/2.4.1/AddOrReplaceReadGroups.jar I=sample1.bam O=sample1.Gr.bam LB=Whatever PL=Illumina PU=Whatever SM=sample1
samtools/1.3.1/samtools sort sample1.Gr.bam sample1.Gr.sorted
samtools/1.3.1/samtools index sample1.Gr.sorted.bam

Example (using demo files):

$ mkdir SNP
$ cd ..
$ bowtie2 -x ASU/ref_98 -1 BCpc/1M_PM-KNE10.1.fq -2 BCpc/1M_PM-KNE10.2.fq -S SNP/1M_PM-KNE10.sam

$ cd SNP
$ samtools view -bS 1M_PM-KNE10.sam > 1M_PM-KNE10.bam
$ java -jar picard.jar ValidateSamFile.jar INPUT= 1M_PM-KNE10.bam
$ java -jar picard.jar AddOrReplaceReadGroups.jar I=1M_PM-KNE10.bam O=1M_PM-KNE10.Gr.bam LB=Whatever PL=Illumina PU=Whatever SM=1M_PM-KNE10
$ samtools sort 1M_PM-KNE10.Gr.bam 1M_PM-KNE10.Gr.sorted.bam
$ samtools index 1M_PM-KNE10.Gr.sorted.bam

Do this for each of the three demo samples.

13) SNP calling with GATK
For the "Unified Genotyper" module, run the scripts below in the "BCpc" folder:

java -jar gatk/3.4.0/GenomeAnalysisTK.jar -T UnifiedGenotyper -R ref_98.fa -I sample1. Gr.sorted.bam -I sample2.Gr.sorted.bam -I sample3.Gr.sorted.bam -dcov 1000 -glm BOTH -o raw_SNPs_unified.vcf

Example (using demo files):

$ java -jar gatk/3.4.0/GenomeAnalysisTK.jar -T UnifiedGenotyper -R ../ASU/ref_98.fa -I 1M_PM-KNE10. Gr.sorted.bam -I 1M_PM-MD20.Gr.sorted.bam -I 1M_PM-OK.Gr.sorted.bam -dcov 1000 -glm BOTH -o raw_SNPs_unified.vcf

For the "Haplotype Caller" module:

a) Run the script below for each sample:

java -jar gatk/3.4.0/GenomeAnalysisTK.jar -T HaplotypeCaller -R ../ASU/ref_98.fa -I sample1.Gr.sorted.bam --genotyping_mode DISCOVERY -stand_emit_conf 10 -stand_call_conf 30 --minPruning 1 --emitRefConfidence GVCF -o sample1.g.vcf

b) Then run the script below to get SNP data for all samples:

java -jar gatk/3.4.0/GenomeAnalysisTk.jar -T GenotypeGVCFs -R ../ASU/ref_98.fa --variant sampel1.g.vcf --variant sample2.g.vcf --variant sample3.g.vcf -o raw_SNPs_haplo.vcf

14) Filter the GATK output
Select only biallelic SNPs and filter on allele frequency (AF). Run the scripts below in the "BCpc" folder.

java -jar gatk/3.4.0/GenomeAnalysisTK.jar -T SelectVariants -R ref_98.fa -o biallelic_raw_SNPs_unified.vcf --variant raw_SNPs_unified.vcf -restrictAllelesTo BIALLELIC
java -jar gatk/3.4.0/GenomeAnalysisTK.jar -T SelectVariants -R ref_98.fa -V biallelic_raw_SNPs_unified.vcf --filterExpression "AF <= 0.100 || AF >= 0.900" --filterName "AF_0.100_0.900" -o AF_biallelic_raw_SNPs_unified.vcf

> Example (using demo files):
> *$ java -jar gatk/3.4.0/GenomeAnalysisTK.jar -T SelectVariants -R ../ASU/ref_98.fa -o biallelic_raw_SNPs_unified.vcf --variant raw_SNPs_unified.vcf -restrictAllelesTo BIALLELIC*
> *$ java -jar gatk/3.4.0/GenomeAnalysisTK.jar -T SelectVariants -R ../ASU/ ref_98.fa -V biallelic_raw_SNPs_unified.vcf --filterExpression "AF <= 0.100 || AF >= 0.900" --filterName "AF_0.100_0.900" -o AF_biallelic_raw_SNPs_unified.vcf*

S4. Further processing of SNP markers: SNP filtering, SNP consolidation and removal of cosegregating markers.

15) Remove adjacent SNPs
Copy scripts "SNPs_ISL.pl" and "Rm_adj_SNPs.pl" into the "BCpc" folder, and run the script below.

perl SNPs_ISL.pl input_file_vcf

The following output file is generated:
"more_than_one_SNPs_for_same_chromosome_number_in_same_line.vcf"

Run the second script on the output file:
perl Rm_adj_SNPs.pl more_than_one_SNPs_for_same_chromosome_number_in_same_line.vcf

16) Consolidate SNPs
Place the GATK output SNP .vcf file and the python script '*SNP_genotyper.py*' in the same folder.

To consolidate SNPs, run *'SNP_genotyper.py*' by double clicking it (Windows/MacOS) or by typing '*python3 SNP_genotyper.py*' (Linux/UNIX). '*SNP_genotyper.py*' will translate the GATK results from read numbers to genotype (A, B, H, C, D or -), consolidate all SNPs within a GBS tag (if a GBS reference is used) or within a physical distance of 500 bp (if a genome assembly is used as reference), and give the consolidated SNP a unique name 'Tag_' plus number. The output is a tab delimited text file (*.txt) that can be opened with excel.

The '*SNP_genotyper.py*' script will ask for the following questions upon running.

1) 'Enter the GATK .vcf file name: '
    Enter the full name (including extension name) of the GATK output vcf file
2) 'Did you apply any GATK filters (AF/QD/..) on your results? If yes, enter 'y', else, enter 'n' (use lower case only): '

Enter 'y' or 'n' (in lower case)

3) 'Enter the percentage of missing data you want to remove, integer only: '

   For example, if the total accession number is 100, and the input number is 20, all SNP loci with missing data in 20 or more accessions will be removed.

4) 'Enter the threshold for H (2/3/4) check instruction for detail):'

   Enter an integer (suggested 2 to 4) to determine heterozygosity. The script takes allele depth (AD) from the GATK output file at each SNP locus for each accession as input to provide a genotypic score (A, B, H, C, D, -). The answer to this question only affects the definition of "H", "C", and "D".

   If 4 is used, AD is converted to A, B, H, C and D scores as follows:

   $AD_{ref(erence\ allele)}/AD_{alt(ernate\ allele)}$ ratio ≥10: A (homozygous for the Parent 1 allele)

   AD_{ref}/AD_{alt} ≤0.10: B

   $10 > AD_{ref}/AD_{alt} > 4$: D (ambiguous A or heterozygous (H))

   $0.25 > AD_{ref}/AD_{alt} > 0.1$: C (ambiguous B or H).

   All other ratios: H

   If 3 is used, AD is converted to A, B, H, C and D scores as follows:

   $AD_{ref(erence\ allele)}/AD_{alt(ernate\ allele)}$ ratio ≥10: A (homozygous for the Parent 1 allele)

   AD_{ref}/AD_{alt} ≤0.10: B

   $10 > AD_{ref}/AD_{alt} > 3$: D (ambiguous A or heterozygous (H))

   $0.33 > AD_{ref}/AD_{alt} > 0.1$: C (ambiguous B or H).

   All other ratios: H

   If 2 is used, AD is converted to A, B, H, C and D scores as follows:

   $AD_{ref(erence\ allele)}/AD_{alt(ernate\ allele)}$ ratio ≥10: A (homozygous for the Parent 1 allele)

   AD_{ref}/AD_{alt} ≤0.10: B

   $10 > AD_{ref}/AD_{alt} > 2$: D (ambiguous A or heterozygous (H))

   $0.50 > AD_{ref}/AD_{alt} > 0.1$: C (ambiguous B or H).

   All other ratios: H

   Increasing the parameter from 2 to 4 increases the number of loci scored as "H" (definition range increases from ½-2 to ¼-4) and decreases the number of loci scored as "C" (definition range decreases from 1/10 - 1/2 to 1/10 -1/4) and "D" (definition range decreases from 2-10 to 4-10).

   We recommend 4 as input.

5) 'Enter the threshold for sequence depth for robust SNP scoring (*e.g.* 8), integer only: '

   If the number is set to 8, only SNPs with a read depth ≥8 will be scored. If read depth is <8, SNP scores will be listed as missing data ("-"). For populations containing heterozygotes, we recommend setting this number to 8.

17) Select SNPs based on parental scores (only applies to some mapping populations)

    To select SNPs that are 'A' in parent 1 and 'B' in parent 2 (or *vice versa*), run '*SNP_selectByParents.py*' on the '*SNP_genotyper.py*' output file. The script will ask for the input file name, output filename, parents' name, and parents' genotype for selection.

18) Remove cosegregating SNP markers

Place the GATK output SNP .vcf file and the python script '*SNP_cosegregation.py*' in the same folder.

To remove cosegregating markers, run the script '*SNP_cosegregation.py*' in the same way as '*SNP_genotyper.py*'. This code generates two output files; file 1 comprises the SNPs for downstream analyses), and the second file contains information on all cosegregating markers (file name is 'info_' plus file 1 name). In the second output file, the cosegregating markers group together and groups are separated by a blank line. For each cosegregating group, the first marker is the best marker, and this marker will be represented in file 1.

This script can be applied after '*SNP_genotyper.py*', or it can be run independently from '*SNP_genotyper.py*'. If run independently, the file input format must be:

1) Tab delimited file.
2) First row must be the sample name and contain the string "CHROM" anywhere you like.
3) Only A/B/C/D/H/- should be used in the data area, all other strings are not acceptable.

The script will ask the following questions upon running.

1) 'Enter the SNP file name (Tab delimited file *.txt): '
   Enter the input file name; usually this is the output file from '*SNP_genotyper.py*'
2) 'Enter the output file name: '
   Enter a name for the output file; the script will generate two files, one will be the name you enter, the other will be 'info_' plus this name.
3) 'Enter the sample start column number (count start from 1, input 11 if applied on the output of '*SNP_genotyper.py*'): '
   If the input is the '*SNP_genotyper.py*' output, enter 11; else, count the column in which the genotypic data starts, starting from 1.

References:

1. Andrews S. FastQC: a quallity control tool for high throughput sequence data. 2010.
2. Catchen JM, Amores A, Hohenlohe P, Cresko W, Postlethwait JH. Stacks: building and genotyping loci *de novo* from short-read sequences. G3 (Bethesda, Md). 2011;1(3): 171-182. Epub 2012/03/03. doi: 10.1534/g3.111.000240. PubMed PMID: 22384329; PubMed Central PMCID: PMCPMC3276136.
3. Magoc T, Salzberg S. FLASH: Fast length adjustment of short reads to improve genome assemblies. Bioinformatics. 2011;27. Epub 2963.
4. Langmead B, Salzberg SL. Fast gapped-read alignment with Bowtie 2. Nat Meth. 2012;9: 357-359.
5. McKenna A, Hanna M, Banks E, Sivachenko A, Cibulskis K, Kernytsky A, et al. The Genome Analysis Toolkit: A MapReduce framework for analyzing next-generation DNA sequencing data. Genome Res. 2010;20(9): 1297-1303. doi: 10.1101/gr.107524.110. PubMed PMID: PMC2928508.
