# Supplementary material for: UGbS-Flex, a novel bioinformatics pipeline for imputation-free SNP discovery in polyploids without a reference genome: finger millet as a case study
Source: BMC Plant Biol. 2018 Jun 15;18:117. doi: 10.1186/s12870-018-1316-3 (PMC6003085; doi:10.1186/s12870-018-1316-3)
Supplement: Supplementary file 2 — Figure S1. The ‘Bcraw’ folder comprises the raw sequencing files for individual samples. After trimming, the trimmed sequence files are placed in the ‘BCpc’ folder. Using files in the ‘BCpc’ folder as input, all files with equal-length reads are placed in ‘BCfin’ folder. The ‘ASU’ folder holds the ASU method results for all files present in the ‘BCfin’ folder. The ASU results are used to generate a reference; the filtered reference is placed in the ‘Ref’ folder. The trimmed sequences in the ‘BCpc’ folder are aligned (with Bowtie) against the reference files in the ‘Ref’ folder; alignments are used for SNP calling (using GATK); all results are stored in the ‘SNP’ folder. The ‘Process’ number corresponds to the step number in Additional file 1: Data S1. (PPTX 52 kb) [file 12870_2018_1316_MOESM2_ESM.pptx]

## Slide 1
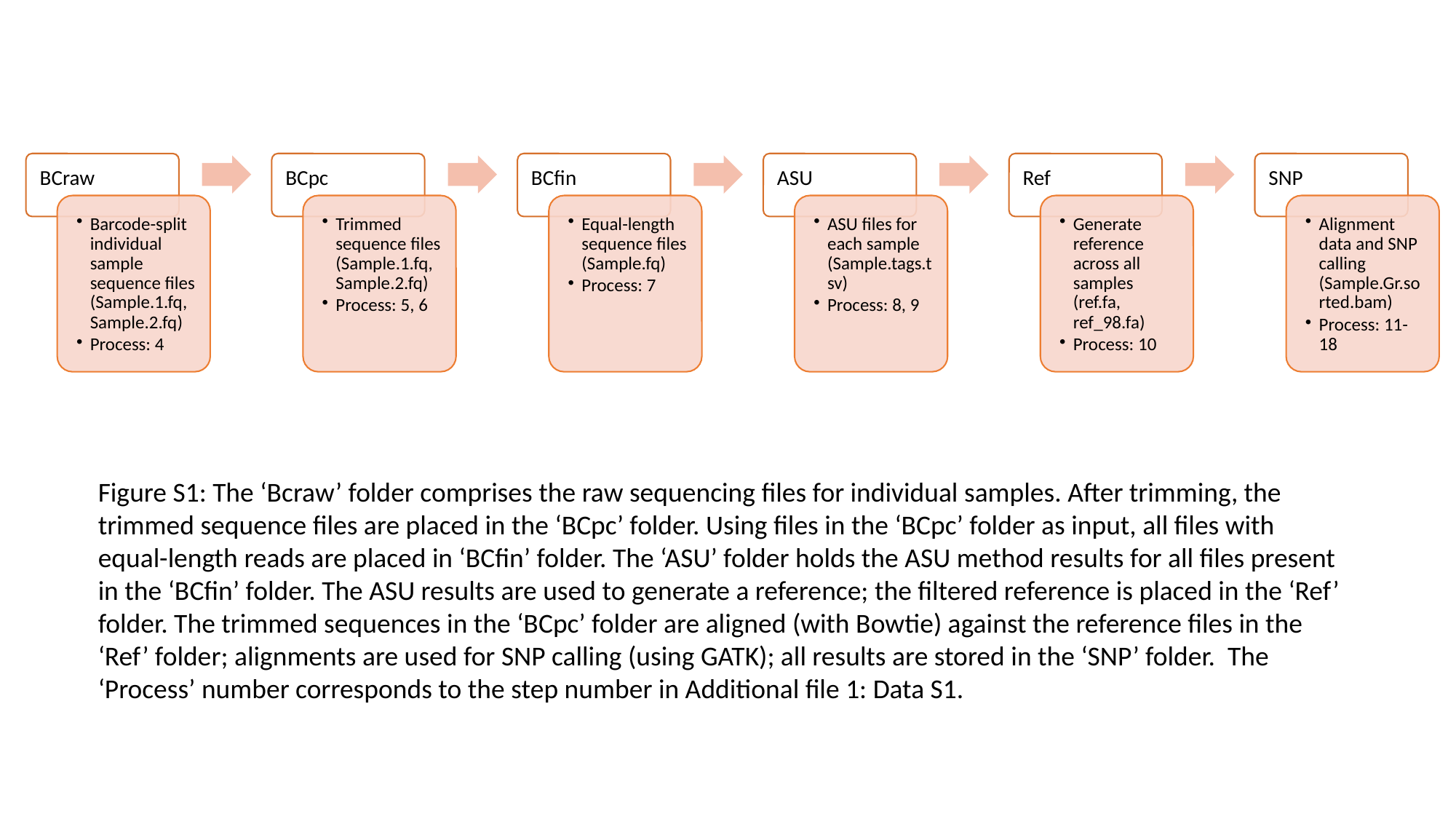

Figure S1: The ‘Bcraw’ folder comprises the raw sequencing files for individual samples. After trimming, the trimmed sequence files are placed in the ‘BCpc’ folder. Using files in the ‘BCpc’ folder as input, all files with equal-length reads are placed in ‘BCfin’ folder. The ‘ASU’ folder holds the ASU method results for all files present in the ‘BCfin’ folder. The ASU results are used to generate a reference; the filtered reference is placed in the ‘Ref’ folder. The trimmed sequences in the ‘BCpc’ folder are aligned (with Bowtie) against the reference files in the ‘Ref’ folder; alignments are used for SNP calling (using GATK); all results are stored in the ‘SNP’ folder. The ‘Process’ number corresponds to the step number in Additional file 1: Data S1.
