# Supplementary material for: UGbS-Flex, a novel bioinformatics pipeline for imputation-free SNP discovery in polyploids without a reference genome: finger millet as a case study
Source: BMC Plant Biol. 2018 Jun 15;18:117. doi: 10.1186/s12870-018-1316-3 (PMC6003085; doi:10.1186/s12870-018-1316-3)
Supplement: Supplementary file 4 — Table S2. Average read depth (across three accessions tested) of GBS reference tags common to all three accessions in the PstI/NdeI fragment pool. (DOCX 13 kb) [file 12870_2018_1316_MOESM4_ESM.docx]

**Table S2: Average read depth (across three accessions tested) of GBS reference tags common to all three accessions in the *Pst*I/*Nde*I fragment pool**

| Read number | % reference tags common to ‘cstacks’ and ‘ASustacks’ | % reference tags uniquely identified with ‘ASustacks’ | Average Read depth across all reference tags | Av. Read depth across reference tags common to ‘cstacks’ and ‘ASustacks’ | Av. Read depth across reference tags uniquely identified with ‘ASustacks’ |
| --- | --- | --- | --- | --- | --- |
| 1,000,000 | 91.0 | 9.0 | 31.9 | 30.8 | 43.5 |
| 2,000,000 | 73.1 | 26.9 | 55.3 | 46.3 | 78.0 |
| 3,000,000 | 57.0 | 43.0 | 78.1 | 52.3 | 109.7 |
