# Supplementary material for: UGbS-Flex, a novel bioinformatics pipeline for imputation-free SNP discovery in polyploids without a reference genome: finger millet as a case study
Source: BMC Plant Biol. 2018 Jun 15;18:117. doi: 10.1186/s12870-018-1316-3 (PMC6003085; doi:10.1186/s12870-018-1316-3)
Supplement: Supplementary file 5 — Table S3. Number and percentage of ApeKI sites present in PstI/MspI and PstI/MspI + ApeKI digests. (DOCX 12 kb) [file 12870_2018_1316_MOESM5_ESM.docx]

Table S3: Number and percentage of *Ape*KI sites present in *Pst*I/*Msp*I and *Pst*I/*Msp*I + *Ape*KI digests

| Enzyme combination | Accession (sample) | Number of reads with *Ape*KI site^1^ | Percentage reads with *Ape*KI site^1^ | Average^2^ percentage of trimmed reads with *Ape*KI site^1^ | Percentage of GBS reference tags^3^ with *Ape*KI site^1^ |
| --- | --- | --- | --- | --- | --- |
| *Pst*I/*Msp*I | KNE 796 | 415,503 | 41.6 | 40.2 | 42.4 |
| *Pst*I/*Msp*I | MD-20 | 367,854 | 36.8 |  |  |
| *Pst*I/*Msp*I | Okhale-1 | 423,489 | 42.3 |  |  |
| *Pst*I/*Msp*I + *Ape*KI | KNE 796 | 213,191 | 21.3 | 19.3 | 0.3 |
| *Pst*I/*Msp*I + *Ape*KI | MD-20 | 153,887 | 15.4 |  |  |
| *Pst*I/*Msp*I + *Ape*KI | Okhale-1 | 210,609 | 21.1 |  |  |

^1^ In subset of 1 M reads

^2^ Average across three accessions

^3^ GBS tags present in all three accessions
