# Supplementary material for: UGbS-Flex, a novel bioinformatics pipeline for imputation-free SNP discovery in polyploids without a reference genome: finger millet as a case study
Source: BMC Plant Biol. 2018 Jun 15;18:117. doi: 10.1186/s12870-018-1316-3 (PMC6003085; doi:10.1186/s12870-018-1316-3)
Supplement: Supplementary file 6 — Figure S2. Comparison of the number of SNPs identified using different SNP callers (UG = Unified Genotyper; HC=Haplotype Caller) and different GBS references (Ref50: tags present in ≥50% of the samples; Ref70: tags present in ≥70% of the samples; Ref50_98: tags present in ≥50% of the samples and only 1 tag retained for tags with ≥98% homology; Ref70_98: tags present in ≥70% of the samples and only 1 tag retained for tags with ≥98% homology. (PPTX 7261 kb) [file 12870_2018_1316_MOESM6_ESM.pptx]

## Slide 1
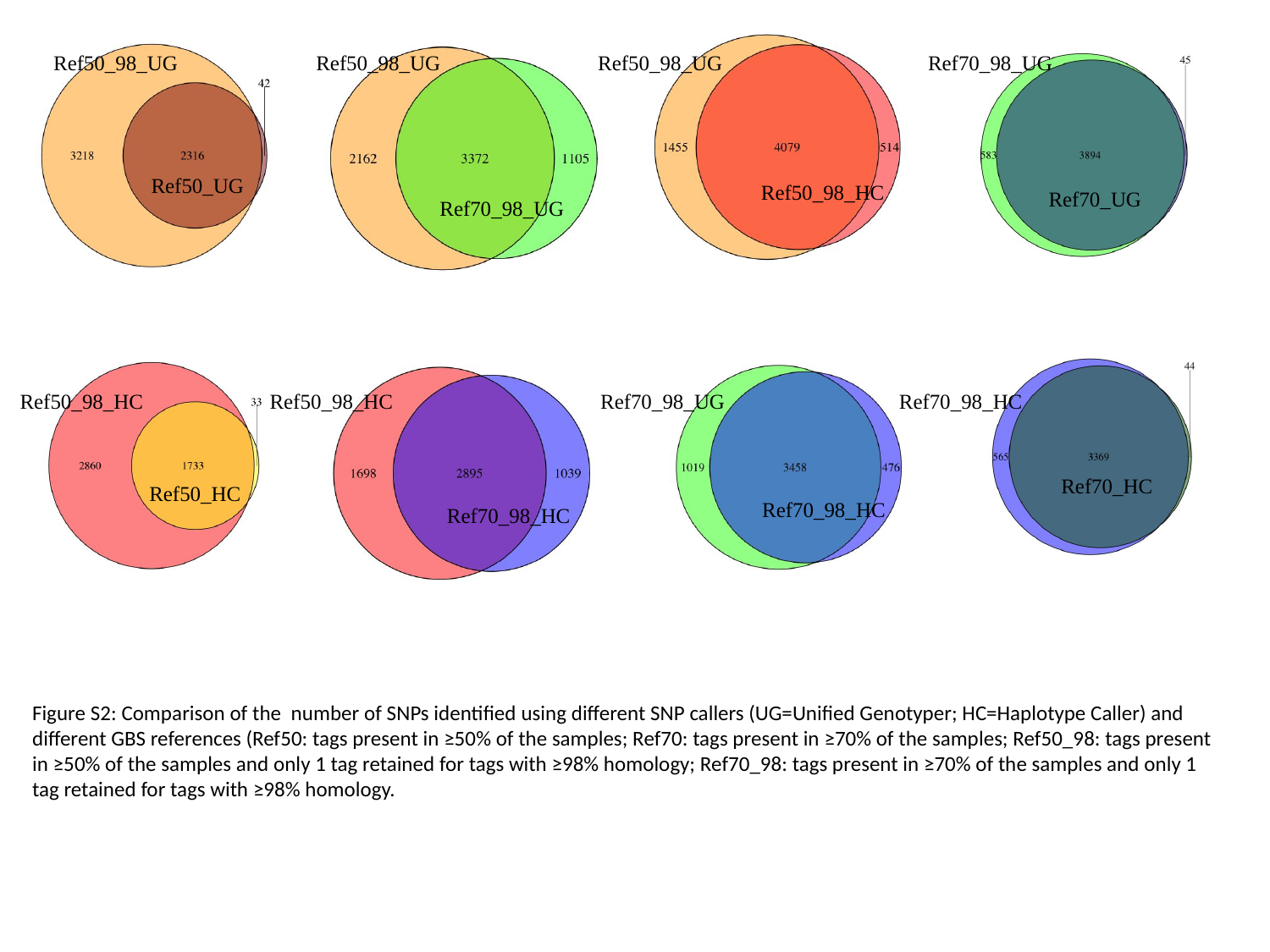

Ref50_98_UG
Ref50_98_UG
Ref50_98_UG
Ref70_98_UG
Ref50_UG
Ref50_98_HC
Ref70_UG
Ref70_98_UG
Ref50_98_HC
Ref50_98_HC
Ref70_98_UG
Ref70_98_HC
Ref70_HC
Ref50_HC
Ref70_98_HC
Ref70_98_HC
Figure S2: Comparison of the number of SNPs identified using different SNP callers (UG=Unified Genotyper; HC=Haplotype Caller) and different GBS references (Ref50: tags present in ≥50% of the samples; Ref70: tags present in ≥70% of the samples; Ref50_98: tags present in ≥50% of the samples and only 1 tag retained for tags with ≥98% homology; Ref70_98: tags present in ≥70% of the samples and only 1 tag retained for tags with ≥98% homology.
