# Supplementary material for: UGbS-Flex, a novel bioinformatics pipeline for imputation-free SNP discovery in polyploids without a reference genome: finger millet as a case study
Source: BMC Plant Biol. 2018 Jun 15;18:117. doi: 10.1186/s12870-018-1316-3 (PMC6003085; doi:10.1186/s12870-018-1316-3)
Supplement: Supplementary file 10 — Figure S3. Effect of the presence of a deletion in a sample relative to the GBS reference allele. A gapped alignment is formed and the 3′ end extends beyond the junction of the forward and reverse reads in the GBS reference resulting in the calling of a SNP at that position. The Integrative Genomics Viewer (Robinson et al. 2011, Nature Biotechnology 29: 24–26; Thorvaldsdóttir et al. 2013, Briefings in Bioinformatics 14: 178–192) was used for visualization. (PPTX 75 kb) [file 12870_2018_1316_MOESM10_ESM.pptx]

## Slide 1
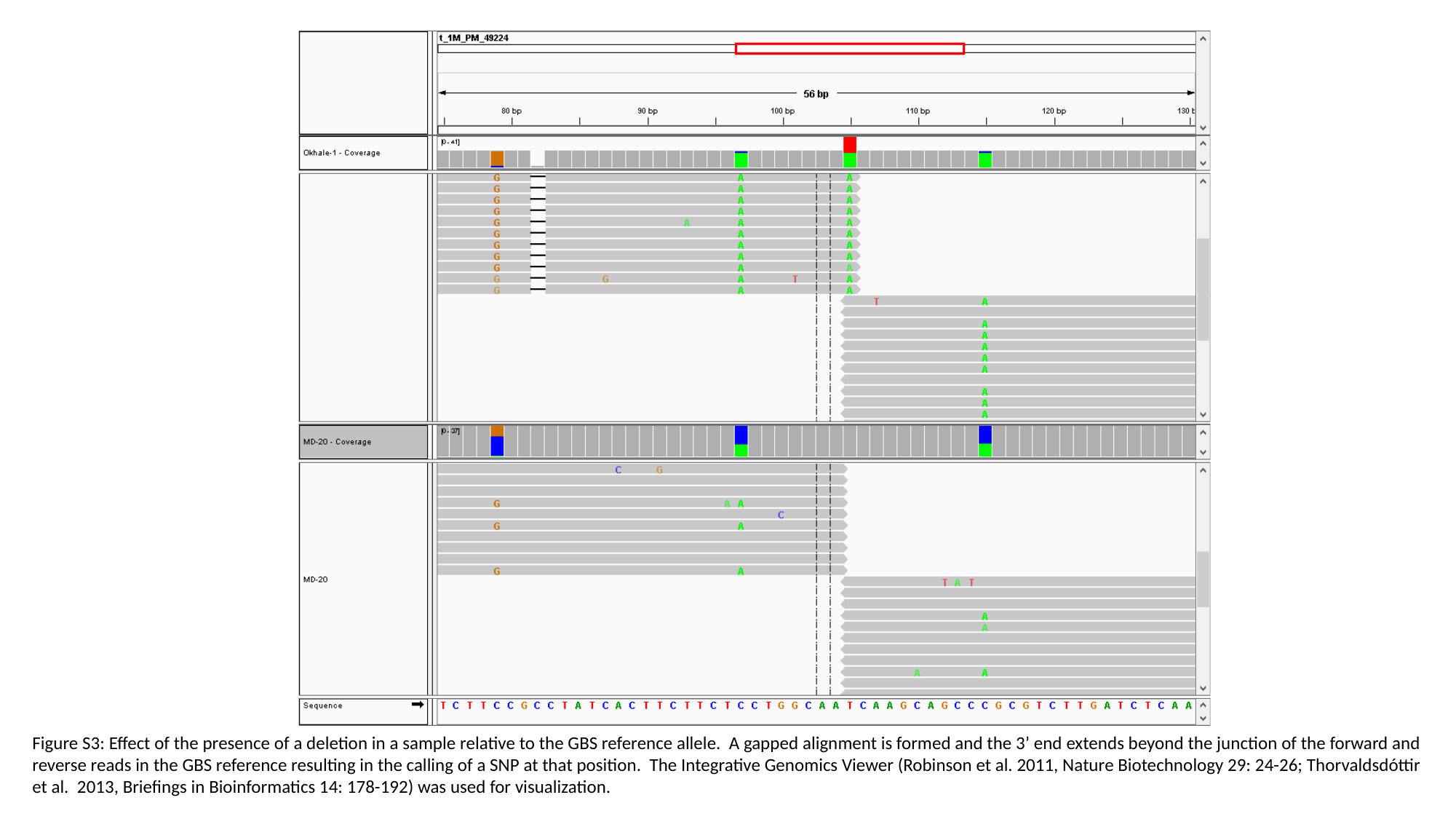

Figure S3: Effect of the presence of a deletion in a sample relative to the GBS reference allele. A gapped alignment is formed and the 3’ end extends beyond the junction of the forward and reverse reads in the GBS reference resulting in the calling of a SNP at that position. The Integrative Genomics Viewer (Robinson et al. 2011, Nature Biotechnology 29: 24-26; Thorvaldsdóttir et al. 2013, Briefings in Bioinformatics 14: 178-192) was used for visualization.
