# Supplementary material for: UGbS-Flex, a novel bioinformatics pipeline for imputation-free SNP discovery in polyploids without a reference genome: finger millet as a case study
Source: BMC Plant Biol. 2018 Jun 15;18:117. doi: 10.1186/s12870-018-1316-3 (PMC6003085; doi:10.1186/s12870-018-1316-3)
Supplement: Supplementary file 11 — Figure S4. Comparison of genetic maps generated using MSTmap (left-hand side) and MAPMAKER (right-hand side). Nearly 65% of markers were reordered in MAPMAKER compared to MSTmap maps. The markers that occupied a different relative position in the two maps are connected by a line. (PPTX 170 kb) [file 12870_2018_1316_MOESM11_ESM.pptx]

## Slide 1
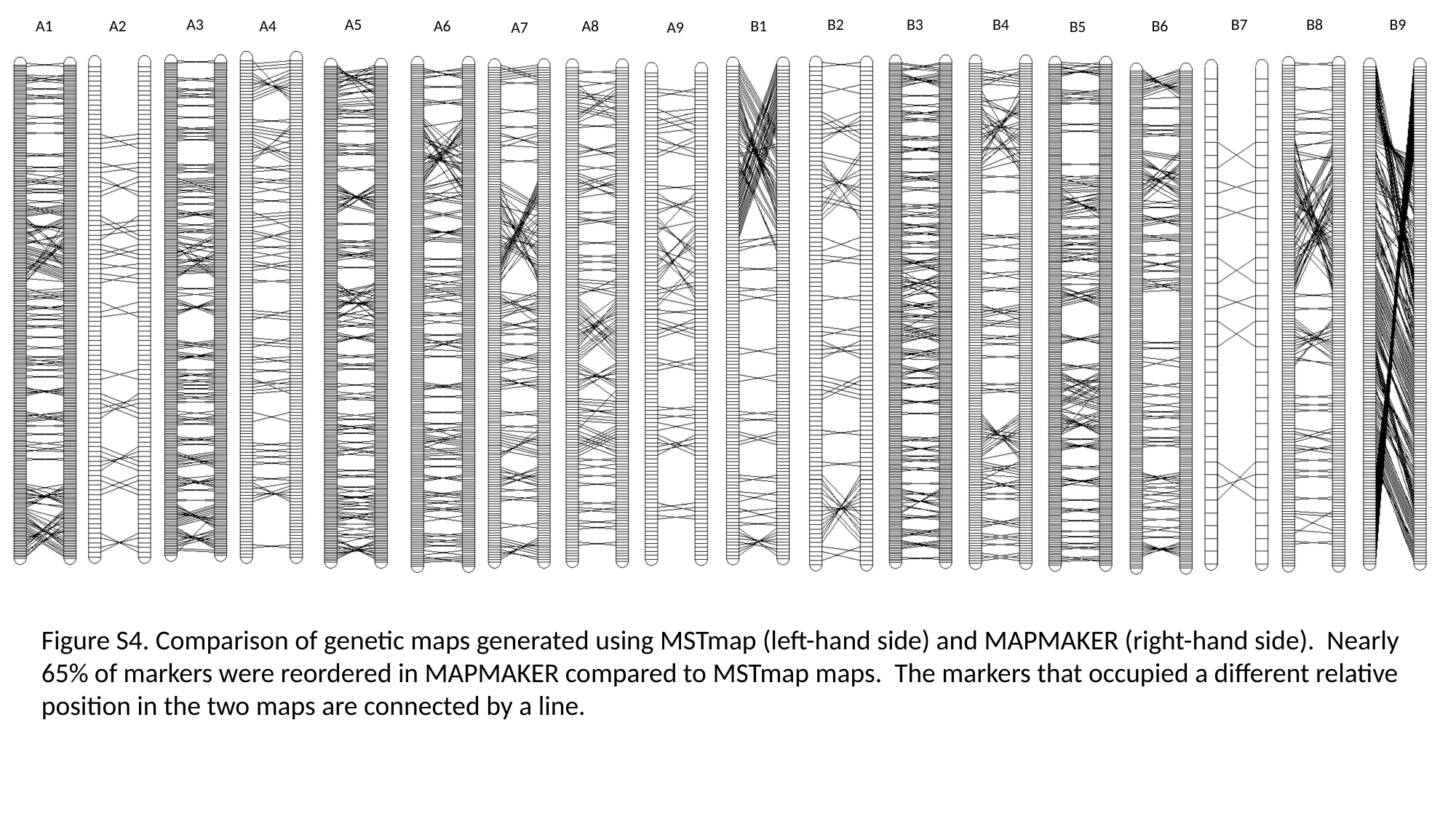

A3
B2
B3
B4
B7
B9
B8
A5
A1
A2
A4
A6
B1
B6
A8
B5
A7
A9
Figure S4. Comparison of genetic maps generated using MSTmap (left-hand side) and MAPMAKER (right-hand side). Nearly 65% of markers were reordered in MAPMAKER compared to MSTmap maps. The markers that occupied a different relative position in the two maps are connected by a line.
